# The Role of Airway Epithelial Cell Alarmins in Asthma

**DOI:** 10.3390/cells11071105

**Published:** 2022-03-24

**Authors:** Christiane E. Whetstone, Maral Ranjbar, Hafsa Omer, Ruth P. Cusack, Gail M. Gauvreau

**Affiliations:** Department of Medicine, McMaster University, Hamilton, ON L8N 3Z5, Canada; whetstoc@mcmaster.ca (C.E.W.); ranjbm1@mcmaster.ca (M.R.); omerh@mcmaster.ca (H.O.); cusackr@mcmaster.ca (R.P.C.)

**Keywords:** airway epithelium, alarmin cytokines, TSLP, IL-33, IL-25, asthma

## Abstract

The airway epithelium is the first line of defense for the lungs, detecting inhaled environmental threats through pattern recognition receptors expressed transmembrane or intracellularly. Activation of pattern recognition receptors triggers the release of alarmin cytokines IL-25, IL-33, and TSLP. These alarmins are important mediators of inflammation, with receptors widely expressed in structural cells as well as innate and adaptive immune cells. Many of the key effector cells in the allergic cascade also produce alarmins, thereby contributing to the airways disease by driving downstream type 2 inflammatory processes. Randomized controlled clinical trials have demonstrated benefit when blockade of TSLP and IL-33 were added to standard of care medications, suggesting these are important new targets for treatment of asthma. With genome-wide association studies demonstrating associations between single-nucleotide polymorphisms of the TSLP and IL-33 gene and risk of asthma, it will be important to understand which subsets of asthma patients will benefit most from anti-alarmin therapy.

## 1. Introduction

Epithelial cell-derived mediators including the alarmin cytokines IL-25, IL-33, and thymic stromal lymphopoietin (TSLP) have emerged as key players in propagating asthma pathogenesis. Release of these alarmins from the airway epithelium of asthmatics leads to the downstream production of type 2 cytokines, most notably IL-4, IL-5, and IL-13 from multiple effector cells. With upregulation of type 2 cytokine expression, many allergic mechanisms are initiated, including eosinophilic inflammation, immunoglobulin (IgG) class switching to IgE, stimulation of B-cell growth, goblet cell metaplasia, and subsequent mucous production. As a result of emerging evidence, IL-25, IL-33, and TSLP are important mediators of inflammation during allergic disease and may prove to be key targets for therapeutic intervention. This review focuses on the role of alarmins in the pathogenesis of asthma and the associations of polymorphisms frequently found in patients.

**Figure 1:**
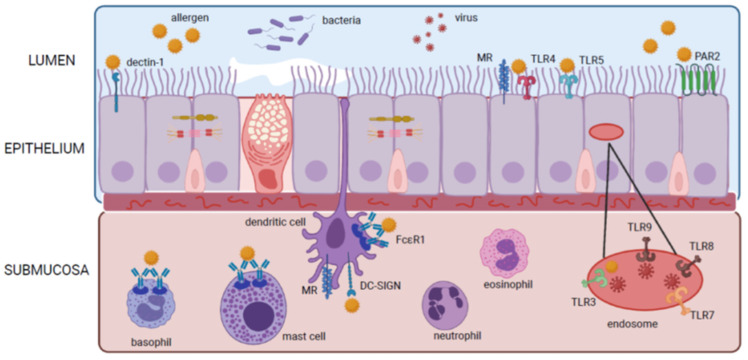
Mechanisms of alarmin cytokine release from epithelium and leukocytes in the airway.

Airway epithelium of the upper (nasal) and lower (lung) airways are the first line in defense against environmental triggers such as allergens, viruses, pollutants, and microbes. These epithelial cells express transmembrane or intracellular pattern recognition receptors (PRRs) that recognize highly conserved microbial motifs called pathogen-associated molecular patterns (PAMPs) and host-derived molecular damage-associated molecular patterns (DAMPs). PRRs recognize invading threats and initiate host defence through the rapid production alarmin cytokines. As part of innate immunity, PRRs can also act as helper proteins for transmembrane receptors, provide opsonization for phagocytosis, direct microbial killing, and initiate the cascade of innate and adaptive immune responses. These cell-bound receptors are divided into two groups: secreted receptors including Toll-like receptors (TLRs), C-type lectin receptors (CLR), protease-activated receptors (PAR), and cytosolic DNA receptors such as AIM2-like receptors (ALRs), and intracellular cytosolic receptors consisting of NOD-like receptors (NLRs), and RIG-I-like receptors (RLRs) [1,2]. As of present, studies have shown that allergens and viruses can interact directly with TRLs, CLRs, and PARs on epithelial and dendritic cells inducing the release of epithelial alarmins to prompt Th2 biased inflammation (Figure 1).

TLRs, RLRs, and NLRs all recognize different but overlapping microbial components; however, only TLRs have been proven to respond to environmental allergen provocation. TLRs are the most extensively researched PPRs, and to date, 10 members of the human TLR family have been identified. Certain TLRs are expressed on the cell surface (TLRs 1, 2, 4–6, and 10), while others (TLRs 3, 7–9) are found in intracellular compartments, such as endosomes. TLRs are expressed on a variety of cells including macrophages, dendritic cells, B cells, specific types of T cells, and structural non-immune cells including fibroblasts and epithelial cells. TLRs can be divided into several subfamilies on the basis of which PAMPs the receptors recognize; TLR1, TLR2, TLR4, and TLR6 recognize lipids, whereas TLR3 and TLR7–9 recognize nucleic acids, and TLR5 recognizes extracellular bacterial flagellin [2,3]. Of particular interest to the development of allergic diseases are lipopolysaccharides (LPS) and peptidoglycans (PGN) found within most natural allergens such as house dust mite (HDM). Recent studies have established the role of TLR4 molecules in the recognition of LPSs, forming a receptor complex including the LPS-binding protein, CD14, MD2, and TLR4 molecules on immune and non-immune cells [4,5]. Among LPSs and PGNs is Der p2, a lipid-binding protein that shares significant structural similarities with MD2 [6,7]. Der p2 is the main allergen component of HDM, and this structural similarity allows for the formation of the receptor complex to be recognized by TLR4. The structural similarity between Der p2 and MD2 explains the mechanistic action of allergic inflammation in response to HDM; however, this is the only allergen with data showing this relationship. Although there is controversy over TLR4′s pro or anti-allergic responses to allergens in human studies, it should be noted that provisional data suggest that TLR1/2/6 [8,9], TLR3 [10], and TLR5 [11] are involved in pro-allergic responses to OVA challenge in experimental mouse models of asthma. TLR4 stimulation by allergens triggers the production of the epithelial-derived alarmins TSLP, IL-33, and IL-25, which support the expansion of Th2 inflammation through the modulation of DC function [6,12]. Aside from allergens, allergic inflammation in the lung can be induced by viruses. The possible mechanism for this viral induction is the activation of TLR3 via viral double-stranded DNA [10,13]. Viral infection was also found to initiate alarmin expression and subsequent Th2 biased allergic inflammation through additional TLRs present on immune cells that detect dsDNA including TLR7, TLR8, and TLR9 [14,15,16,17].

Emerging research suggests an important role for complex carbohydrates in initiating Th2 inflammation against allergens and parasites. CLRs possess a transmembrane PRR with a carbohydrate-binding domain enabling the unique ability to recognize glyo-allergens. A variety of inhaled allergen extracts are known to contain carbohydrates that activate CLRs expressed on dendritic cells and airway epithelial cells including dendritic-cell-specific-ICAM3 (DC-SIGN), macrophage galactose-type C-type lectin receptor (MGL), mannose receptor (MR), and work in conjunction with TLR4 pathways to drive Th2 immune responses [18,19,20]. Specifically, HDM extracts are known to be detected by dectin-1 on epithelial cells [20], whereas HDM, pollen, and dog allergen extracts are detected by DC-SIGN [19,21,22], and HDM, cockroach, dog, and cat allergen extracts are detected by MR on epithelial and DCs [23,24]. No data exist to demonstrate the direct release of alarmins from CLRs recognition of allergen extracts; however, Al-Ghouleh et al. demonstrated TSLP secretion by epithelial cells upon stimulation and recognition of HDM by TLRs was in some part carbohydrate-dependent [25].

Furthermore, there is increasing interest in the role protease-activated receptors (PAR) play in the pathogenesis of asthma. PAR are G protein-coupled receptors activated by proteolytical cleavage of the amino-terminus, unmasking endogenous tethered ligands for the receptor binding pockets [26]. PAR-2 has been shown to be expressed on structural cells including endothelial and epithelial cells; fibroblasts; and immune cells such as lymphocytes, monocytes, mast cells, neutrophils, eosinophils, macrophages, and DCs. PAR-2 can be activated by serine proteases from allergen sources such as HDM, cockroach, pollen, and mold [27,28]. Protease detection through PAR-2 in airway epithelial cells leads to epithelial alarmin release, cytokine production, cell detachment, and morphological changes in airway epithelial cells, all contributing to an allergic response [29,30,31,32].

## 2. Epithelial Alarmins in Asthma

### 2.1. IL-25

#### 2.1.1. IL-25 Expression

IL-25 (also known as IL-17E) was originally identified by Fort et al. as a Th2 producing cytokine, implicated in the induction of the Th2-like response [33]. IL-25 belongs to the IL-17 family that comprises six members: IL-17A, IL-17B, IL-17C, IL-17D, IL-25, and IL-17F. IL-25 shares 16% homology with IL-17A and has distinct biological effects that differ from other IL-17 family members [33,34]. Eosinophils and basophils have been described as the major source of IL-25 in patients with asthma, but a wide range of cells produce and secrete IL-25, including also epithelial/endothelial cells, activated Th2 cells, alveolar macrophages, bone marrow-derived mast cells, and fibroblasts [35,36,37,38,39,40,41].

#### 2.1.2. IL-25 Receptor Expression and Signaling

While IL-25 is extensively expressed by many cell types, the IL-25 receptor (IL-25R) expression is more restricted. IL-25 has receptors on innate immune cells including invariant natural killer T (iNKT) cells, ILC2s, eosinophils, basophils, mast cells, and antigen-presenting cells (APCs) (Table 1).

The IL-25 receptor is expressed by the cell in a form of disulphide-linked dimers, composed of IL-17RA and IL-17RB subunits, of which both contain a conserved SEFIR domain at the cytoplasmic region [62,63]. IL-25 binds directly to the IL-17RB subunits and further associates with the IL-17RA subunit to form a complex that is required to mediate a downstream signaling cascade [64]. The IL-17RA subunit is shared by receptors of several IL-17 cytokine family members including IL-17A and IL17R (when paired with IL-17RC) and IL-17 (when paired with IL-17RE) [65], while IL-17RB can bind with lower affinity to IL-17B [34,66,67]. IL-25 interaction with the IL-17RA/IL-17RB receptor complex with co-stimulatory molecules CD3 and CD28 result in the upregulation of several transcription factors including nuclear factor kappa B (NF-kB) and p38 mitogen-activated protein kinases (MAPKs), c-Jun amino-terminal kinase (JNK), STAT5, GATA3, and NF-ATC1. This upregulation attributes to the secretion of IL-4, IL-5 IL-6, IL-13 CXCL10, CXCL9, and CCL5.

Upon ligand binding, IL-25R recruits the adaptor molecule Act1 through the homotypic interactions of the SEFIR domains, also present on Act1. Once IL-25 is bound to the adaptor protein Act1 (also known as CIKS), Act1 mediates multiple signaling pathways important to immune response and cell fate decisions [68,69,70,71,72]. Both NF-kB and MAPK (JNK and p38) induce transcriptional activation in an Act1-dependent manner [48,73,74]. Additionally, Act1 ubiquitinates TNF-receptor-associated factor (TRAF) adaptors that are recruited to the receptor complex and play a critical role in signal transduction [75]. TRAF6 is crucial in IL-25R-mediated activation of the NF-kB pathway as NF-kB activation is blocked by a dominant-negative form of TRAF6; however, TRAF6 does not affect the activation of MAPK [71,74,76,77]. TRAF4 amplifies IL-25-mediated signaling by activating the E3 ligase smadubiquitin regulatory factor 2, leading to the ubiquitylation and subsequent degradation of the inhibitory protein, deleted in azoospermia DAZ-associated protein 2 (DAZAP2) [78,79]. Interestingly, TRAF2, TRAF3, and TRAF5 are unnecessary for IL-25 signaling but prove to be critical in IL-17A-mediated signaling [74,78,80,81]. IL-25 also activates Janus kinase/single transducer and activator of transcription (JAK/STAT), which prove to be essential for survival and transcriptional activation in an Act1-independent manner [82,83].

Janus kinase/signal transducers and activators of transcription signal (JAK/STAT) are responsible for the induction of a multitude of cellular mechanisms. Four JAK and seven STAT molecules have been identified in humans. Typically, JAK activation results in the phosphorylation of STATs; however, STATs can also be activated via JAK family-independent mechanisms [84]. STAT5 is recruited to the IL-25 receptor in a TRAF4-dependent, Act1-independent manner through direct interaction with tyrosine residues Y444 and Y454 on the IL-17RB subunit [70,85]. It is theorized that TRAF4-smadubiquitin regulatory factor 2-dependent degradation of DAZAP2 following the IL-25 stimulation facilitates the phosphorylation of Y444 and Y454 by JAKs, thus leading to the recruitment of STAT5 to the IL-17RB subunit [78]. However, exact molecular mechanisms have yet to be identified. STAT5 activation via its cooperation with GATA-3 is sufficient to generate a Th2 profile [55,56,57].

#### 2.1.3. The Role of IL-25 in the Airways

IL-25 is expressed in airway epithelium as a preformed cytokine and stored in the cytoplasm, thereby permitting rapid release upon cell stimulation by environmental triggers, including allergens. IL-25, both protein and transcript, is found in higher levels in airways of asthmatic patients compared to controls [59,86]. Allergic asthmatic patients with late asthmatic responses (bronchoconstriction, AHR, eosinophilic airway inflammation) to inhaled allergen challenge have further elevations of IL-25 in the epithelium and submucosa, and in eosinophils and basophils post-challenge [38,45,46,87], as well as elevated IL-17RB in myeloid dendritic cells (mDCs), plasmacytoid dendritic cells (pDCs), eosinophils and their progenitors, and basophils measured in blood and sputum [45,46,47,88], thereby linking IL-25 signaling to acute worsening of asthma. In addition, asthmatic patients with higher IL-25 transcript levels were shown to have more severe disease [86], suggesting this cytokine is an important driver of asthma.

IL-25 induces Th2-skewed inflammation marked by the over expression of cytokines IL-4, IL-5, and IL-13, and in asthmatic patients, this leads to an increase in serum IgE levels, blood eosinophilia, and pathological changes in the lungs characterized by increased mucus production and epithelium cell hyperplasia [64]. ILC2 rapidly proliferate in response to IL-25 [89] and production of IL-25 by epithelial cells induces chemokines including TARC, eotaxin, and macrophage-derived chemokine (MDC), thereby playing a role in in airway remodeling and angiogenesis [59], which contributes to asthma severity. In patients with allergic diseases such as allergic asthma, local cells in the affected tissue, including eosinophils, basophils, mast cells, and keratinocytes, express both IL-25 and IL-25R, and further drive an allergic response [40]. IL-25 directly enhances Th2 cytokine production from TSLP-DC-activated Th2 memory cells by enhancing the production of Th2 transcription factors in an IL-4-independent manner [40]. Additionally, pDC expression of TLR9, FcεR1, and CD68 can be regulated by IL-25, suggesting that IL-25 may act as a link between adaptive and innate immune responses through its ability to control TLR9 expression and TLR9–induced responses [88].

IL-25 release by airway epithelial cells contributes to many other pathogenic features of asthma, including the recruitment of eosinophils, airway mucus over secretion, and airway remodeling. IL-25 activation of eosinophils upregulates the gene expression and release of various chemokines including MCP-1, MIP-1alpha, and cytokines IL-8 and IL-6 [48]. Additionally, IL-25 was shown to significantly upregulate the surface expression of ICAM-1 while suppressing ICAM-3 and L-selectin on eosinophils, thereby facilitating endothelial transmigration [49].

In basophils, activation of IL-17RB by IL-25 was shown to inhibit basophil apoptosis and augment IgE-mediated basophil degranulation of inflammatory mediators including histamine, LTC_4_, IL-3, and IL-13 [44]. IL-25 alone has limited effects on ILC2s; however, in combination with co-stimulatory cytokines such as IL-2, it promotes the phosphorylation of GATA3 resulting in the rapid production of type 2 cytokines [50]. Mast cells are shown to express equal levels of IL-25R to eosinophils, T cells, and endothelial cells [38]; however, the exact mechanisms by which IL-25 stimulates mast cells is not known. Although macrophages are potent producers of IL-25, these cells also respond to IL-25 released from lung epithelial cells by downregulating their Rab27a and Rab27b expression, resulting in the suppression of exosome release and attenuating exosome-induced TNF-alpha expression and secretion from neighboring macrophages [42]. Thus, IL-25 acts in a negative feedback fashion, providing crosstalk between epithelial cells and macrophages.

In addition to innate immune cells expressing IL-25R, a variety of adaptive immune cells including Th2 and Th9 cells are responsive to IL-25. Th2 cells are shown to have the most sustained IL-25R expression, and IL-25R is highly expressed on CD4+ Th2 memory cells [40]. IL-25 activation of CD4+ T cells mediates enhanced type-2 immune response through activation of naïve peribronchial lymph nodes, with CD3 and CD8 cells increasing the production of IL-4, IL-5, and IL-13 but not INF-alpha [53,54]. IL-25 co-stimulation of Th2 memory cells enhances their Th2 polarization and cytokine production in an IL-4 independent manner [40]. Surprisingly, IL-9-producing Th9 cells were found to have enhanced IL-17RB expression when treated with IL-4 and tumor growth factor beta (TGF-β). This suggests that Th9, an inhibitor of Th2 differentiation, responds to IL-25, and that the dual expression of IL-17RB on Th9 and Th2 potentially functions as a feedback loop to regulate Th2 cells and their response to IL-25 [58].

Structural cells including endothelial cells also respond to IL-25, forming a positive feedback loop in the airways [38]. IL-25 was found to contribute to angiogenesis by increasing endothelial cell VEGF/VEGF receptor [59]. Fibroblasts [61] and airway smooth muscle cells (ASMC) also express IL-25R under specific conditions [60]. Fibroblasts constitutively express IL-25R, which is further increased when stimulated with TNF-alpha and decreased with TNF-beta1 to induce and maintain eosinophilic inflammation. IL-25 upregulates CC chemokine ligand (CCL) 5 and CCL11, and synergistically induces GM-CSF and CXC chemokine ligand (CXCL) 8 with TNF-alpha stimulation [61]. IL-25 can also modulate bronchial airway smooth muscle hyperplasia and collagen deposition. ASMC expression of IL-25R is upregulated by TNF-alpha and downregulated by INF-gamma. In addition, stimulation with IL-25 elevates expression of extracellular matrix components (ECM) (mainly procollagen-aI and lumican mRNA) from ASMC, indicting a possible role of airway remodeling through the induction of ECM [60].

Overexpression of IL-25 in the lung in mice induces allergic TH2 responses [35] and IL-25 blockade attenuates allergen-induced AHR and type 2 inflammation in the lungs of sensitized mice [90]. Together these observations strongly support IL-25 as a critical factor for allergic responses, and that IL-25 blockade has the potential to alleviate asthma and allergic inflammation. There has only been one randomized clinical trial testing the efficacy of IL-25 blockade, and this study used an anti-IL-17RA monoclonal antibody, brodalumab, in a population of moderate to severe asthmatics. Treatment with brodalumab failed to show improvement of lung function measured by peak flow, or symptoms measured by Asthma Control Questionnaire in the overall patient population [91]. However, a subgroup of patients with high bronchodilator reversibility demonstrated a clinically meaningful response to brodalumab, suggesting blockade of IL-25 may indeed be beneficial in some asthma patients. Experiments in mice have shown that combined blockade of IL-25 together with IL-33 and TSLP was significantly better at inhibiting OVA-induced Th2 inflammation, suggesting IL-25 blockade alone may not provide sufficient protection. 

### 2.2. IL-33

#### 2.2.1. IL-33 Expression

IL-33 is a member of the IL-1 cytokine family, being most homologous to IL-1-beta and IL-18, and it is constitutively expressed in the nuclei of cells including endothelial cells, fibroblast reticulate cells from secondary lymphoid tissues, keratinocytes, airway smooth muscle cells, and epithelial cells [92,93,94,95,96,97]. IL-33 is synthesized as a full-length (pro-IL-33) precursor, consisting of a non-classical nuclear localization sequence and a chromatin-binding domain at the N-terminus and a C-terminal domain with cytokine activity [98], giving IL-33 the unique ability to function as a cytokine and as a transcriptional regulator. Localization of IL-33 in the nucleus appears to regulate IL-33 cytokine activity, suggesting that IL-33 sequestration to the nucleus functions to limit its pro-inflammatory capabilities [99,100].

#### 2.2.2. IL-33 Receptor Expression and Signaling

IL-33 binds to a heteromeric receptor. IL-33 was first identified as the ligand for suppression of tumorigenicity 2 (ST2, also referred to as IL-1RL1, T1, or IL-33R) [101], which had been characterized as an orphan receptor important in type 2 responses within the lungs [102,103]. ST2 is encoded by IL1RL1 gene and is a member of the Toll-like/IL-1-receptor superfamily [104,105]. ST2 has two main splice forms resulting from differential promoter binding. The transmembrane isoform (ST2L) acts as the receptor for IL-33 [101] while the soluble isoform (sST2) lacks the transmembrane domain and acts as a decoy receptor that blocks IL-33 activity [106,107]. ST2L is highly expressed on hematopoietic cells and mature leukocytes including mast cells, Th2 lymphocytes, macrophages, basophils, eosinophils, and ILC2s [101,108,109,110,111,112,113,114] (Table 2). To induce signaling, IL-33 also binds to IL-1 receptor accessory protein (IL1RAcP), the second protein of the IL-33 heteromeric receptor.

Receptor signaling utilizes a range of transcription factors. Upon binding of IL-33 with ST2L, the target cells undergo conformational changes, resulting in the recruitment of IL1RAcP forming an IL-33/ST2/IL-1RAcP complex. Formation of this complex causes rapid activation of the NF-kB and MAPK (ERK1/2), JNK, p38, JAK2, SYK, and phosphophoinositied-3-kinase (PI3K)/protein kinase B (AKT) signaling pathways, and the IRAK (IL-1R-associated kinase)/TRAF6 module [111,113,114,134,135] to promote the production and release of a range of proinflammatory mediators including IL-6, IL-8, MCP-1, and MCP-3 [130,136]. The TRAF6 module induces a cascade of events by further activating TAK1 (MAP3K7) and causing activation of the transcriptional regulator NF-kB, which in turn activates stress-activated protein kinase p38 and c-Jun N-terminal kinase (JNK). Activation of ERK signaling has also been reported but appears to be independent of TRAF6-mediated signaling [137]. The induction of IL-6 and IL-13 production by IL-33 relies heavily on MK2/3-mediated activations of ERK1/2 and PI3K signaling [118].

Activation of signaling modules by IL-33 also occurs in a cell-type specific manner to induce the variety of downstream effector proteins, but in a diverse range of cell types. With relevance to asthma, the p38 MAPK pathway is important. Activation of p38 MAPK induces the phosphorylation and subsequent binding of GATA3 to the IL-5 and IL-13 promotor regions within ILC2 cells [128]. IL-33 also activates MAP-kinases, in particular p38, and the NF-kB pathway to stimulate eosinophils [113]. IL-33 induces other pathways such as the PI-3 kinase/AKT/mTOR pathway in murine cells including Th2, ILCs and eosinophils, and human endothelial cells [129]. In mast cells, the cross-activation of c-Kit by ST2 results in the phosphorylation of c-Kit at Y721 and phosphorylation of ERK1/2, JNK1, PKB, and STAT3 [118], whereas in macrophages and primary human monocyte-derived macrophages, IL-33 activates the ERK1/2, JNK, and PI3K-Akt signaling to decrease the expression of ADAMTS family of metalloproteases [110]. IL-33 induces production of IL-13 from mast cells via a MyD88-5-/12-LO-BLT2-NF-kB cascade [112]. This broad range of signaling pathways across a wide variety of cell types serves to amplify the pro-inflammatory scope of IL-33.

#### 2.2.3. Role of IL-33 in Airways

IL-33 is a central component for activation of both the innate and adaptive arms of immunity, and in this capacity release of IL-33 into the airways plays an important role in the pathobiology of asthma. A myriad of cells found in asthmatic airways express IL-33. IL-33 is highly expressed in bronchial epithelium of patients with asthma [87] with increased expression after exposure to environmental triggers such as allergen [87,138]. Furthermore, proteolytic maturation of IL-33 by allergen is proposed to be a mechanism for induction of allergic inflammation [139]. Bronchial epithelium also expresses ST2L, and this co-expression of ligand and receptor is thought to amplify the inflammatory response in a positive feedback loop [133].

In the lower airways, the release of IL-33 has been proposed to be responsible for the development and exacerbation of airway hypersensitivity and asthma [140,141]. IL-33 is one of the earliest cytokines released in response to allergens [128] and is elevated in the lung epithelium [95], airway smooth muscle [141], and bronchoalveolar lavage [142], correlating with disease severity [96]. IL-33 and sST2 levels are markedly elevated in blood and sputum of patients with eosinophilic asthma [143,144], and in those experiencing acute exacerbations [144]. Exposure to inhaled allergen in mild allergic asthmatic subjects enhances ST2L expression on eosinophils in blood and sputum, and this increased receptor expression can be replicated by in vitro stimulation of eosinophils with IL-33 [145]. ILC2s are major cellular targets of IL-33, and upon activation by IL-33, their production of Th2 cytokines plays a critical role in type-2 immunity and eosinophil homeostasis [125].

IL-33 has been shown to be responsible for inducing early immune development and polarization toward type 2 T cell inflammation [101,146] through activation of resident dendritic DC to become mature and induce DC-stimulated differentiation of naïve CD4+ T cells into polarized type 2 T cells [111,115,116] that produce cytokines such as IL-5 and IL-13 [101]. In turn, cytokine release from type 2 T cells prolongs eosinophil survival, adhesion, and degranulation [123], thereby contributing to asthma pathogenesis. IL-33 activated DCs also prolong survival, enhance adhesion, and stimulate cytokine production of mast cells [117], as well as stimulate secretion of IL-13 by alveolar macrophages [109]. The mechanistic pathways through which IL-33 activated DCs induce type 2 immunity in humans is complex and not fully understood.

IL-33 can also induce cell activation, independent of DCs. IL-33 directly induces cytokine production, including IL-4, IL-5, IL-13, and IL-9, in CD4+ T cells [121]. Memory Th2 cells express even higher levels of ST2 than effector Th2 cells [130,131]. Interestingly, Th2 cells also negatively regulate IL-33-mediated cytokine production. In the presence of a-galactosylceramine antigen presentation, IL-33 dose-dependently increases the production of IL-4 and INF-gamma in iNKTs, while IL-33 stimulation in combination with IL-12 induces INF-gamma production from iNKT and from NK cells [119]. Macrophages express both the ST2L and sST2; IL-33 stimulation induces naïve macrophages to produce M1 chemokines such as CCL3 and enhances the expression of M2 chemokine markers including CCL17, CCL18, and CCL24 in previously polarized macrophages [108,109]. Potent activation of ILC2s is achieved through upregulating co-stimulatory molecules OX40L and PD-L1, thereby inducing ILC2s to produce IL-5 and IL-13 [114,126]. On a per cell basis, IL-33 is able to induce approximately 10-fold more IL-5 and IL-13 from ILC2s compared to activated type 2 T cells [127], indicating the relative potential contribution of each cell to type 2 inflammation. IL-33 promotes basophil IgE-dependent and IgE-independent release of histamine, as well as secretion of IL-4, IL-5, IL-6, IL-8, IL-9, IL-13, MCP, and MIP [113,119,120,121,122]. IL-33 also induces basophil CD11b expression, adhesion, and prime eotaxin-induced migration [113]. Moreover, IL-33 induces eosinophil CD11b expression, adhesion to albumin, fibronectin, ICAM-1, and VCAM-1, as well as enhancing eosinophil survival and cytokine production [124,125].

The best evidence for the IL-33/ST2 axis playing a pathogenic role in asthma comes from the results of randomized clinical trials. Blockade of IL-33 with itepekimab, compared to placebo, was shown to reduce exacerbations in patients with moderate-to-severe asthma when treatment with steroids was withdrawn [147], and blockade of ST2 with astegolimab reduced the annualized asthma exacerbation rate in a broad population of severe asthma patients, including those defined as eosinophil-low [148]. Importantly, this study highlights the role of IL-33 in inflammation driven by non-T2 pathways.

### 2.3. Thymic Stromal Lymphopoietin

#### 2.3.1. TSLP Expression

TSLP is a member of the IL-2 family of cytokines and was initially identified as a pre-B cell growth factor. TSLP is a distant paralog of IL-7, as evidenced by sharing a common receptor subunit IL-7Ra. In humans TSLP has two main isoforms—a short isoform is expressed under basal conditions, and a longer isoform is induced by inflammatory stimuli [149,150,151]. Cleavage of TSLP by serine proteases may regulate TSLP protein levels and/or functions [152]. TSLP is expressed at barrier surfaces where both exogenous and endogenous danger signals are sensed and cause its release from a variety of cells including epithelial cells, epidermal keratinocytes, fibroblasts, myeloid dendritic cells, macrophages, basophils, and monocytes [151,153,154,155,156,157,158,159,160,161,162].

#### 2.3.2. TSLP Receptor Expression and Signaling

The effects of TSLP are exerted through binding to the high-affinity TSLP receptor (TSLPR) complex that consists of a TSLP-binding chain and the IL-7Ra subunit [163,164]. The TSLPR is broadly expressed within hematopoietic cell populations including mDCs, CD4+ and CD8+ T cells, regulatory T cells (Treg), B cells, mast cells, NKT cells, monocytes, CD34+ progenitor cells, eosinophils, basophils, and airway smooth muscle [165,166,167] (Table 3).

TSLPR binds with high affinity to TSLP. While IL-7Ra does not bind to TSLPR alone with measurable affinity, IL-7Ra binds to the TSLP/TSLPR complex with high affinity, making this binary assembly mechanistically crucial for effective signal transduction [201,202]. Interestingly, TSLPR and IL-7Ra have some affinity for each other in the absence of TSLP, suggesting that a preformed receptor–receptor TSLP-mediated complex may be required under certain conditions [201].

Binding of TSLP to the IL-7Ra/TSLP receptor complex phosphorylates STAT-3 and STAT-5 through JAK1/JAK2 and PI3K pathway signaling [168,203,204,205]. Other studies have shown that TSLP can also induce STAT-1 phosphorylation in CD4+ T cells and myeloid DCs [177,178]. TSLP-mediated signaling via the JAK/STAT pathways has been extensively studied in DCs and T lymphocytes [177,179,180]. In mDCs, potent and long-lasting activation of JAK1 and JAK2 by TSLP induces the phosphorylation of mitogen-activated protein kinase (MAPK)s, extracellular signal-regulated kinase (ERK), and c-Jun N-terminal kinase (JNK) [172]. STATs play a crucial role for TSLP-DC signaling, and JAKs (JAK1/2) can be activated following stimulation by TSLP and initiate signaling pathways downstream via STATs; however, which subtype of STATs are involved and JAKs’ specific role in this process remains unconfirmed [172,176].

DCs are activated by TSLP and CD40L, along with stimuli that bind to TLRs (including LPS, polyI:C, and R848). TSLP promotes DC maturation and upregulation of MHC class II, as well as the co-stimulatory molecules CD40, CD86, CD54, CD80, CD83, and CD-LMAP on human mDCs [170]. However, unlike CD40L and the TLR ligands, TSLP does not stimulate but rather limits the production of pro-inflammatory cytokines TNF-a, IL-1B, and IL-6 and the Th1-polarizing cytokines IL-12 and type 1 IFNs [156,206]. TSLP priming of DCs promotes the upregulation of OX40L, which induces naïve T cells to acquire an inflammatory Th2-like phenotype with the production of type 2 cytokines including IL-4, IL-5, IL-13, and TNF-alpha, but not regulatory cytokines such as IL-10 [171]. TSLP simulation of DCs and subsequent Th2 polarization has been shown to involve the Notch pathway when human bronchial epithelial cells are exposed to environmental pollutants [181]. Recent reports also show the induction of the tight junction protein claudin-7 following TSLP stimulation of DCs was dependent on the NF-kB pathway [182]. TSLP can also induce DCs to produce chemotactic factors including IL-8, eotaxin-2, TARC/CCL17, MDC/CCL22, and I-309/CCL1 that participate in the recruitment of Th2 cells, eosinophils, and neutrophils to the airways [156,172,173]. Additionally, TSLP promotes a priming effect on DCs to induce the expansion and functionality of CRTH2+ CD4+ Th2 memory cells [166,174,175], which in human CD4 T-cell cultures can drive the differentiation of Tregs and hinder the developments of FOXP3+ Tregs [166,174,175]. TSLP can also suppress the production of IL-10 by pulmonary Tregs [197].

#### 2.3.3. TSLP in the Airways

Functional studies have demonstrated that TSLP can strongly induce the development, expansion, and effector function of immune cells, inducing the innate and adaptive immune system. In response to pathogenic stimuli or mechanic injury, TSLP exacerbates allergic inflammation by activating many effector cells that participate in the immune cascade, including ILC2s and myeloid DCs [169,207,208,209,210].

As a key cell in the innate immune system, the mDC responds to TSLP by upregulating co-stimulatory molecules CD40, CD80, CD86, and OX40L that drive induction of Th2 cells and Th9 cells, as well as their elaboration of type 2 cytokines [156,170,173,179,194,211,212,213,214,215,216]. TSLPR has also been described in other innate cells such as macrophages [169] and NK cells [184]. The role of TSLP stimulation of DCs is well characterized compared to that of monocytes/macrophages. It has recently been shown that TSLP also induces CD80 expression in human peripheral blood CD14+ monocytes/macrophages, indicating monocyte/macrophage activation [165]. Additionally, TSLP was associated with an increase in the percentages of CD14^dim/−^, CD80+, CD11c+, and HLA-DR+ cells, being consistent with the increased differentiation into myeloid DCs [165]. TSLP induces the release of T cell-attracting chemokines (TARC)/CCL17, DC-CK1/pulmonary and activation-regulated chemokine (PARC)/CCL18, macrophage-derived chemokine (MDC)/CCL22, and MIP3β/CL19 [168]. TSLP can also drive the differentiation and activation of alternatively activated macrophages (referred to as M2 macrophages) during allergic inflammation [169].

In some asthma models, TSLP-driven allergic inflammation is mediated by ILCs. There is an important role for the TSLP/ILC axis since TSLP has been shown to mediate resistance to corticosteroids in ILC2s examined from human PBMCs and bronchoalveolar lavage BAL fluid [217]. Human NKT cells were shown to induce the production of IL-4 and IL-13 in the presence of TSLP stimulation [188], as well as the addition of DCs to the culture upregulated the IFN-gamma expression [188].

Granulocytes migrate to inflamed airways where they contribute to tissue damage. In eosinophils, the hallmark cell of asthma, TSLP, has been shown to prevent apoptosis; upregulate the adhesion molecule CD18; intercellular adhesion molecule-1; and induce IL-6, CXCL8, CXCL1, and CCL2, and downregulate expression of L-selectin. Collectively, these effects support eosinophil extravasation and migration to sites of inflammation. Human cord CD34+ cells have also been found to express TSLPR, and stimulation of these cells by TSLP causes a dose-dependent release of IL-5, IL-13, and GM-CSF; induces the release of chemokines CCL22, CCL17, CXCL8, and CCL1; and increases the expression of IL-5Ra [189,190].

TSLP receptors have been described on mast cells [153] and basophils [208]. Mast cells that are stimulated by TSLP act synergistically with IL-1B and TNF-alpha to release IL-5, IL-13, IL-6, IL-8, IL-10, GM-CSF, and chemokines CXCL8 and CCL1, while suppressing the release of TGF-beta [153]. Mast cells have also been shown to regulate epithelial TSLP expression [218], suggesting that epithelial-derived TSLP can directly affect mast cell function, and mast cells in turn can regulate epithelial TSLP expression. Basophils are not only a significant source of TSLP, but also a potent TSLP receptor expressing cell [219]. Overnight incubation of basophils with TSLP upregulates expression of receptors for TSLP, IL-33, and IL-25 [45]. TSLP stimulation of basophils also increases their expression of intracellular Th2 cytokines (IL-4, IL-13), increases markers of activation (CD203c), and enhances basophil degranulation. The basophil/TSLP axis is not fully investigated in humans and warrants further study.

TSLP is a powerful regulator of the adaptive immune system. CD4+ T cells and CD8+ T cells generally do not respond to TSLP under resting conditions; however, following adequate activation, TSLP receptor expression increases on the surface of these cells [196]. TSLP signaling directly on naïve T cells in the presence of TCR stimulation promotes the proliferation and differentiation to Th2 cells through the induction of IL-4 gene transcription [179,194,212]. It has long been known a subset of CD4+ T cells described as regulatory T cells (Tregs) can be mediated by TSLP, having TSLP induce proliferation and differentiation via DCs [176,220]. TSLP is also involved in proliferation and differentiation of B-cell progenitors [221], directly supporting B-cell lymphopoiesis [191,192]. In the presence of TSLP, multilineage-committed CD34+ progenitor cells, pro-B cells, and pre-B cells differentiate and proliferate through the phosphorylation of STAT5 [193].

Structural cells of the airways are also responsive to TSLP. Airway smooth muscle cell expression of the proinflammatory cytokines IL-6, as well as the CC/CXC chemokine IL-8 (CXCL8 and eotaxin-1/CCL11), is dependent on stimulation by TSLP and downstream signaling of STAT3 and MAPKs (ERK1/2, p38, and JNK), but not STAT5 [200].

Anti-TSLP therapy has now been approved for treatment of asthma, with tezepelumab demonstrating significant attenuation of allergen-induced asthmatic responses [222] and improvement in asthma exacerbation rate [223,224] through a reduction in blood eosinophils and airway nitric oxide levels. While anti-OX40L monoclonal antibody (mAb) treatment has been shown to moderate Th2 differentiation in murine studies [225], this treatment approach was not successful in a human model of asthma [226].

## 3. Variants of Alarmins Genes in Asthma

Some patients respond better than others to asthma treatments, and it is interesting to consider why this happens. It is generally accepted that genetic and environmental interactions are important risk factors resulting in complications of asthma. The importance of gene variants in altering the severity of this multifactorial disease has been long confirmed by several GWAS studies conducted in the past decade [227,228,229]. One of the important reasons that has drawn attention to conducting genetic polymorphism studies is that not all research findings are similar for a candidate single nucleotide polymorphism (SNP) [230]. Recent genetic evidence has shown associations between certain SNPs in alarmin genes and susceptibility to asthma. Table 4 summarizes the recently studied SNPs of alarmins and their association with asthma.

### 3.1. IL-33 Single-Nucleotide Polymorphisms

Many of the SNPs in the IL-33 gene that have been studied in asthmatic patients have demonstrated a positive and direct association with asthma. Rs3939286, rs16924159, rs1342326, and rs992969 are among the SNPs that are more pronounced in the asthmatic population compared to the healthy subjects [231,233,234]. Reports on the associations of different variants show that the presence of only one mutated allele in the genome can result in a significant direct or inverse association. For instance, rs928413 is a SNP significantly associated with increased risk of asthma when carrying the AA genotype, while individuals with the heterozygous genotype (GA) have a decreased risk of asthma compared to those who do not carry the mutated allele [233]. While most association studies of SNPs focus on the risk and susceptibility of asthma, Tse et al. demonstrated that SNPs rs1342326 and rs7037276 are inversely associated with emergency department failure in children with moderate to severe asthma [232], suggesting those that carry these SNPs may have a better response to common asthma therapy. Findings of genome-wide association studies show that ethnic background can have an essential role in determining the direction of association between different SNPs and disease risk or susceptibility [240]. Therefore, it is important to conduct more association studies in populations with different ethnic backgrounds.

### 3.2. TSLP Single-Nucleotide Polymorphisms

Among the studies conducted on the gene polymorphisms of TSLP gene and asthma, several SNPs have been shown to impact the susceptibility to this disease. Interestingly, one candidate SNP rs2289276 has consistently demonstrated a protective effect, whereby carrying the SNP is associated with a lower risk of asthma [235,236,241]. Furthermore, the lower risk was more pronounced in females, meaning that SNPs can act as sex-specific risk factors [241]. The mechanism behind rs2289276 is not fully understood; however, Harada et al. proposed that the presence of this SNP on the promoter region of the TSLP gene might decrease the binding affinity of transcription factors, which in turn leads to downregulation of TSLP and its inflammatory impacts [151]. While rs2289276 was demonstrated as a SNP with inverse correlations with asthma, another frequently studied SNP rs1837253 is significantly correlated with increased asthma susceptibility. In addition, carrying this SNP may lead to a higher risk of asthma in females [239]. It is not clear how these SNPs exert their impacts in a sex specific manner; however, the effects of sex hormones on the prevalence differences between male and female genders has been confirmed previously [242]. Therefore, the sex-specific patterns of alarmin SNPs could be justified by the alterations that are made in sex hormone production by these gene polymorphisms. Some TSLP SNPs have inconsistent associations, for instance, rs2289278 was reported to have no effects on asthma risk in an Egyptian population [235] but was shown to have significant associations with asthma severity in Iranians [236]. Conflicting results in these studies might be due to low frequency of such SNPs in the studied populations or racial and environmental differences.

## 4. Alarmins and Other Allergic Diseases

In addition to asthma, the epithelial-derived alarmins IL-33, IL-25, and TSLP play important roles in the pathogenesis of other allergic diseases including allergic rhinitis, chronic rhinosinusitis, atopic dermatitis, food allergy, and allergic keratoconjunctivitis. These chronic diseases can affect the quality of life and are more common in industrialized and developed countries [243]. Here, we briefly describe the recent findings regarding the role of alarmin cytokines in the development of these allergic diseases.

### 4.1. Allergic Rhinitis

Allergic rhinitis is one of the most common allergic diseases affecting between 10–30% of the population worldwide and usually diagnosed by its common symptoms: nasal congestion and rhinorrhea. This allergic condition often coexists with asthma, sinusitis, conjunctivitis, and nasal polyposis, suggesting that alarmins may as well function as the pathological factors in these diseases [244]. Studies on the levels of alarmins in patients with allergic rhinitis have shown that levels of IL-33, IL-25, and TSLP are significantly higher compared to healthy controls [245,246,247]. Interestingly, a recent study has discovered the synergistic effects of IL-33 and TSLP produced by nasal epithelia cells in promoting inflammatory cell proliferation and reducing the apoptosis [248].

IL-25, demonstrated to be rapidly released from the cytoplasm of epithelial cells upon exposure to aeroallergens, is shown to increase the proliferation of inflammatory ILC2s, which produce significantly higher levels of IL-17, a cytokine which has been shown to be associated with more severe allergic rhinitis [249].

### 4.2. Atopic Dermatitis

Atopic dermatitis is an inflammatory disease of the skin that is more prevalent in children than adults [250]. Recent studies in human and mouse skin have shown that alarmin cytokines are upregulated in keratinocytes of atopic dermatitis and result in eczema symptoms [251,252,253]. Upregulation of alarmins has also been reported at the gene expression level in a recent study by Griffiths et al., where mRNA analysis of skin biopsy samples of atopic dermatitis patients and healthy individuals has shown that IL-33-related genes are elevated in both lesion and non-lesion skin samples, whereas TSLP genes are upregulated significantly in lesion compared to non-lesion skin [254]. Recent findings on the role of IL-25 in the pathology of atopic dermatitis has shown that patients with moderate and severe atopic dermatitis tend to have higher IL-25 serum concentrations [255]. It has also been reported that exogenous allergens such as house dust mite can prompt the production of IL-25 and lead to more aggravated symptoms in these patients [256].

### 4.3. Food Allergy

Food allergy is an IgE-mediated allergic response to food antigens that is growing in prevalence. While antigens from foods are usually tolerated by the immune system, patients with food allergies suffer from adverse immune responses when exposed to certain food antigens such as peanut, milk, egg, fish, shellfish, wheat, soy, and seeds [257]. The food specific allergic response happens because of the epithelial damage in the gut, which induces the production of alarmins in response antigen activation of ILC2s and DCs, and subsequently promoting the Th2 response similar to the events in other allergic diseases [258]. A study published in 2021 has shown that alarmin levels increase in patients with shrimp allergy compared to healthy participants. However, there was no correlation between the alarmin levels and the severity of allergic reactions in patients when consuming the food [259]. The observed efficacy of anti-IL-33 therapies in clinical trials provides clear evidence that IL-33 is involved in the pathogenesis of food allergy [260].

## 5. Conclusions

The epithelial-derived alarmins IL-25, IL-33, and TSLP are widely recognized as factors released in response to danger signals from the environment; however, many other cell types can also synthesize and release alarmins in inflamed tissue. The alarmin cytokines are known to be key orchestrators of inflammation in allergic diseases by coordinating cellular responses in the innate and adaptive immune systems as well as structural cells. Genome-wide association studies have demonstrated relationships between SNPs and risk of allergic disease, including asthma. Furthermore, the importance of TSLP and IL-33 in the pathogenesis of asthma has been confirmed by studies showing efficacy of drugs that block these cytokines. It remains to be determined if the response to anti-alarmin treatment in patients with allergic disease and improvement in asthma control is related to environmental factors, or to genetic SNPs.

## Figures and Tables

**Table 1:** IL-25 and pathogenic effects in asthmatic airways.

	Cell Type	Functional Effect of IL-25 on Cellular Function
**Innate Immunity**	Alveolar macrophage	↓ Rab27a and Rab27b expression [42]
		↓ Release of exosomes [42]
	DC	↑ Activated Th2 memory cells [40]
		↑ Chemotaxis of IL-9 producing cells [43]
	Mast cell	Receptor expressed but function not defined [38]
	Basophils	↓ Apoptosis [44]
		↑ Histamine degranulation, IL-4, IL-13 [40,44,45]
	Eosinophils	Eosinophil expression of IL-25 receptor [40,46,47]
		↑ MCP-1, MIP-1a, IL-8, IL-6 [48], ICAM-1 [49]
		↓ ICAM-3, L-selectin [49]
		Signaling through JNK, MAPK (p38), NF-kB [48,49]
	ILC2	↑ IL-4, IL-5, IL-13 [50]
		↑ Expression of IL-33R [51]
		Signaling through MAPK (p38) [50]
	Invariant NKT cells	↑ IL-13 [52]
		↑ CCL17, CCL22, C10/CCL6, ECF-L [52]
**Adaptive Immunity**	CD4+ T cell	↑ IL-4, IL-5, IL-13 [40]
		↑ CD3, CD8 cells [53,54]
	Th2 cells	↑ IL-4, IL-5, IL-13 [40]
		Signaling through STAT5 [55,56,57]
	Th9 cells	↑ Inhibit Th2 differentiation [58]
**Structural Cells**	Epithelial/endothelial cells	Receptor expression [38,59]
		↑ Angiogenesis [59]
		↑ Endothelial cell VEGF/VEGF receptor expression) [59]
		Signaling through PI3K/Akt and Erk/MAPK [59]
	Airway smooth muscle cells	↑ TNF-a [60]
		↓ INF-y [60]
		↑ EMC procollagen-aI and lumican mRNA [60]
		Signaling through NF-kB [60]
	Fibroblasts	↑ CCL5, CCL11, GM-CSF, CXCL8 [61]

**Table 2:** IL-33 and pathogenic effects in asthmatic airways.

	Cell Type	Functional Effect of IL-33 on Cellular Function
**Innate Immunity**	Macrophages/monocytes	↑ M2 macrophage polarization [108,109]
		↓ ADAMTS family of metalloproteases [110]
		Signaling through ERK1/2, JNK, and PI3k-Akt [110]
	Dendritic cells	↑ Th2 polarization [111,115,116]
		↑ CD4+ T cell release of IL-5 and IL-13 [101]
		↑ Macrophage release of IL-13 [108]
	Mast cells	↑ Mast cell survival, adhesion, cytokine production [117]
		↑ IL-6, IL-13 [112]
		MK2/3 activation of ERK1/2, PI3k
		c-Kit activation of ERK1/2, JNK1, PKB, and STAT3 [118]
	Basophils	↑ Histamine, IL-4, IL-5, IL-6, IL-8, IL-9, IL-13, MCP, MIP [113,119,120,121,122]
		↑ CD11b expression [113]
		↑ Adhesion and priming of eotaxin-induced migration [113]
		Signaling through ERK1/2, JNK, p38, and NF-kB [113]
	Eosinophils	↑ Eosinophil survival, adhesion, degranulation [123]
		↑ Mature eosinophils and eosinophil progenitors from bone marrow [113]
		↑ Adhesion and survival [124]
		↑ Expression of CD11b [124]
		↑ Il-8 [113,125]
		Signaling through MAPK (p38) and NF-kB [113]
	ILC2s	↑ IL-5, IL-13 [114,126,127]
		Signaling through PI3k/AKT/mTOR, MAPK (p38) [128,129]
	iNKT cells	↑ IL-4, INF-gamma [119]
**Adaptive Immunity**	CD4+ T cells	↑ IL-9 [121]
	Th2 cells	↑ IL-4, IL-5, IL-13 [130,131]
		↓ IL-4, IL-5, IL-13 in certain conditions [132]
		Signaling through PI3k/AKT/mTOR [118,129] and MAPK [132]
**Structural Cells**	Epithelial/endothelial cells	↑ IL-8
		Signaling through ERK and MAPK (p38) [133]

**Table 3:** TSLP and pathogenic effects in asthmatic airways.

	Cell Type	Functional Effect of TSLP on Cellular Function
**Innate Immunity**	Monocytes/macrophages	↑ TARC/CCL17, PARC/CCL18, MDC/CCL22, MIP3β/CL19 [168]
		↑ CD80 [165]
		↑ M2 macrophages [169]
	Myeloid DC	↑ MHC class II, CD40, CD86, CD54, CD80, CD83 [170]
		↑ OX40L [170,171]
		↑ IL-8, eotaxin-2, TARC/CC17, MDC/CCL22, I-309/CCL1 [156,172,173]
		↑ Expansion of CRTH2+ CD4+ Th2 memory cells [166,174]
		↑ Differentiation of Tregs [174,175]
		Signals through Jagged-1, JAK1, JAK2, Akt, ERK, JNK, NF-kB (p50, RelB), STAT1, STAT3, STAT4, STAT5, STAT6 [172,176,177,178,179,180,181,182]
	Mast cells	↑ IL-5, IL-13, IL-6, IL-10, IL-8, GM-CSF [153,183]
		↑ CXCL8, CCL1 [153,184]
		↑ TGF- β [153]
	Basophils	↑ CD69, CD62L, CD11b, CD123, IL-33R, IL-18R surface expression [167]
		↑ IL-4, IL-13 [45]
		↑ CD203c, IL17RB expression [45]
	Eosinophils	↑ Survival, adhesion [185]
		↑ CD18, ICAM-1, CXCL8, CXCL1, CCL2, IL-6 [185]
		↓ L-selectin [185]
		Signals through ERK, p38, NF-kB [185]
	ILC2s	↑ IL-25R, IL-33R expression [186,187]
	Natural killer T cells	↑ IL-4, IL-13 [188]
	CD34+ progenitor cells	↑ Eosinophilopoiesis and basophilopoiesis [189,190]
		↑ IL-5, IL-13, GM-CSF, CCL22, CCl17, CXCL8, CCL1 [189,190]
		↑ IL-5Rα expression [189,190]
**Adaptive Immunity**	B cells	↑ Proliferation [191,192]
		↑ Development [191,192]
		Signals through STAT1, STAT3, STAT5, JAK1, JAK2 [193]
	Th2 cells	↑ Proliferation [156]
		↑ Differentiation [156]
		↑ IL-5, IL-4, IL-13 [156,171]
	CD4+ T cells	↑ Proliferation [156]
		↑ Differentiation [156,191]
		Signals through STAT1, STAT5, JAK1, JAK2 [177,178,179,194,195]
	CD8+ T cells	↑ Proliferation [196]
		Signals through STAT5, Bcl-2 [196]
	T regulatory cells	↓ Development [176]
		↑ Differentiation [166,174,175,176]
		↓ IL-10 [197]
**Structural Cells**	Epithelial/endothelial cells	↑ Airway obstruction mechanisms [198,199]
		Signals through TARC/CCL17, MDC/CCL22, IP-10/CXCL10 [198,199]
	Airway smooth muscle	↑ IL-6, CXCL8, CCL11 [200]
		↑ Migration, actin polymerization, cell polarization [200]
		Signals through STAT3, MAPKs (ERK1/2, p38 and JNK) [200]

**Table 4:** IL-33 and TSLP SNPs and their association with asthma.

Gene	SNP	Alleles	Alternative Allele	Phenotype	N (Case/Control)	Function	Reference
IL-33	rs1342326	A/C	C	Childhood atopic and adult asthma	126/300	Associated with atopic, mild and late-onset asthma, and a higher level of eosinophils in peripheral blood	[231]
		A/C	C	Childhood asthma	491 cases	Associated with decreased odds of ED management failure	[232]
	rs7037276	C/G,T	G,T	Childhood asthma	491 cases	Associated with decreased odds of ED management failure	[232]
		C/G,T	G,T	Adult asthma	104/111	No association between the variant alleles or genotypes and susceptibility to asthma.	[233]
	rs3939286	T/A,C	A,C	Childhood atopic and adult asthma	126/300	Associated with non-atopic and childhood-onset asthma	[231]
	rs928413	G/A,C,T	A,C,T	Adult asthma	104/111	Allele *A* and its homozygous genotype AA were significantly associated with an increased risk to develop asthma, whereas the heterozygous genotype GA was associated with a decreased asthma-risk	[233]
	rs16924159	G/A	A	Adult asthma	104/111	AA genotype was indicated as susceptibility variant to asthma	[233]
	rs992969	A/C,G,T	C,G,T	Adult asthma	13,395 cases	Associated with blood eosinophil levels, asthma, and eosinophilic asthma	[234]
TSLP	rs2289276	C/T	T	Adult asthma	126/300	TT genotype of rs2289276 was inversely associated with the risk of asthma in a sex specific manner	[235]
		C/T	T	Childhood asthma	40/20	TT genotype of rs2289276 was protective to develop asthma	[236]
		C/T	T	Adult asthma	272/398	No association with asthma	[237]
	rs2289278	C/G	G	Adult asthma	126/300	No association with asthma	[235]
		C/G	G	Childhood asthma	40/20	rs2289278 significantly associated with asthma severity	[236]
	rs1837253	C/T	T	Adult asthma	123/100	rs1837253 associated with asthma susceptibility	[238]
		C/T	T	Adult asthma	272/398	No association with asthma	[237]
		C/T	T	Adult asthma	250/250	T allele associated with increased susceptibility to asthma in females	[239]
	rs3806933	C/T	T	Adult asthma	272/398	No association with asthma	[237]

## Data Availability

Not applicable.

## References

[1] Bals R., Hiemstra P.S. (2004). Innate immunity in the lung: How epithelial cells fight against respiratory pathogens. Eur. Respir. J..

[2] Opitz B., van Laak V., Eitel J., Suttorp N. (2010). Innate immune recognition in infectious and noninfectious diseases of the lung. Am. J. Respir. Crit. Care Med..

[3] Chaudhuri N., Dower S.K., Whyte M.K., Sabroe I. (2005). Toll-like receptors and chronic lung disease. Clin. Sci..

[4] Zielen S., Trischler J., Schubert R. (2015). Lipopolysaccharide challenge: Immunological effects and safety in humans. Expert Rev. Clin. Immunol..

[5] Vercelli D. (2008). Discovering susceptibility genes for asthma and allergy. Nat. Rev. Immunol..

[6] Hammad H., Lambrecht B.N. (2008). Dendritic cells and epithelial cells: Linking innate and adaptive immunity in asthma. Nat. Rev. Immunol..

[7] Trompette A., Divanovic S., Visintin A., Blanchard C., Hegde R.S., Madan R., Thorne P.S., Wills-Karp M., Gioannini T.L., Weiss J.P. (2009). Allergenicity resulting from functional mimicry of a Toll-like receptor complex protein. Nature.

[8] Redecke V., Häcker H., Datta S.K., Fermin A., Pitha P.M., Broide D.H., Raz E. (2004). Cutting edge: Activation of Toll-like receptor 2 induces a Th2 immune response and promotes experimental asthma. J. Immunol..

[9] Buckland K.F., O’Connor E., Murray L.A., Hogaboam C.M. (2008). Toll like receptor-2 modulates both innate and adaptive immune responses during chronic fungal asthma in mice. Inflamm. Res..

[10] Torres D., Dieudonné A., Ryffel B., Vilain E., Si-Tahar M., Pichavant M., Lassalle P., Trottein F., Gosset P. (2010). Double-stranded RNA exacerbates pulmonary allergic reaction through TLR3: Implication of airway epithelium and dendritic cells. J. Immunol..

[11] Wilson R.H., Maruoka S., Whitehead G.S., Foley J.F., Flake G.P., Sever M.L., Zeldin D.C., Kraft M., Garantziotis S., Nakano H. (2012). The Toll-like receptor 5 ligand flagellin promotes asthma by priming allergic responses to indoor allergens. Nat. Med..

[12] Willart M.A., Deswarte K., Pouliot P., Braun H., Beyaert R., Lambrecht B.N., Hammad H. (2012). Interleukin-1α controls allergic sensitization to inhaled house dust mite via the epithelial release of GM-CSF and IL-33. J. Exp. Med..

[13] Reuter S., Dehzad N., Martin H., Böhm L., Becker M., Buhl R., Stassen M., Taube C. (2012). TLR3 but not TLR7/8 ligand induces allergic sensitization to inhaled allergen. J. Immunol..

[14] Hatchwell L., Collison A., Girkin J., Parsons K., Li J., Zhang J., Phipps S., Knight D., Bartlett N.W., Johnston S.L. (2015). Toll-like receptor 7 governs interferon and inflammatory responses to rhinovirus and is suppressed by IL-5-induced lung eosinophilia. Thorax.

[15] Beeh K.M., Kanniess F., Wagner F., Schilder C., Naudts I., Hammann-Haenni A., Willers J., Stocker H., Mueller P., Bachmann M.F. (2013). The novel TLR-9 agonist QbG10 shows clinical efficacy in persistent allergic asthma. J. Allergy Clin. Immunol..

[16] Sykes A., Edwards M.R., Macintyre J., Del Rosario A., Gielen V., Haas J., Kon O.M., McHale M., Johnston S.L. (2013). TLR3, TLR4 and TLRs7-9 Induced Interferons Are Not Impaired in Airway and Blood Cells in Well Controlled Asthma. PLoS ONE.

[17] Papaioannou A.I., Spathis A., Kostikas K., Karakitsos P., Papiris S., Rossios C. (2017). The role of endosomal toll-like receptors in asthma. Eur. J. Pharm..

[18] Saunders S.P., Walsh C.M., Barlow J.L., Mangan N.E., Taylor P.R., McKenzie A.N., Smith P., Fallon P.G. (2009). The C-type lectin SIGNR1 binds Schistosoma mansoni antigens in vitro, but SIGNR1-deficient mice have normal responses during schistosome infection. Infect. Immun..

[19] Hsu S.C., Chen C.H., Tsai S.H., Kawasaki H., Hung C.H., Chu Y.T., Chang H.W., Zhou Y., Fu J., Plunkett B. (2010). Functional interaction of common allergens and a C-type lectin receptor, dendritic cell-specific ICAM3-grabbing non-integrin (DC-SIGN), on human dendritic cells. J. Biol. Chem..

[20] Nathan A.T., Peterson E.A., Chakir J., Wills-Karp M. (2009). Innate immune responses of airway epithelium to house dust mite are mediated through beta-glucan-dependent pathways. J. Allergy Clin. Immunol..

[21] Shreffler W.G., Castro R.R., Kucuk Z.Y., Charlop-Powers Z., Grishina G., Yoo S., Burks A.W., Sampson H.A. (2006). The major glycoprotein allergen from Arachis hypogaea, Ara h 1, is a ligand of dendritic cell-specific ICAM-grabbing nonintegrin and acts as a Th2 adjuvant in vitro. J. Immunol..

[22] Emara M., Royer P.J., Mahdavi J., Shakib F., Ghaemmaghami A.M. (2012). Retagging identifies dendritic cell-specific intercellular adhesion molecule-3 (ICAM3)-grabbing non-integrin (DC-SIGN) protein as a novel receptor for a major allergen from house dust mite. J. Biol. Chem..

[23] Royer P.J., Emara M., Yang C., Al-Ghouleh A., Tighe P., Jones N., Sewell H.F., Shakib F., Martinez-Pomares L., Ghaemmaghami A.M. (2010). The mannose receptor mediates the uptake of diverse native allergens by dendritic cells and determines allergen-induced T cell polarization through modulation of IDO activity. J. Immunol..

[24] Emara M., Royer P.J., Abbas Z., Sewell H.F., Mohamed G.G., Singh S., Peel S., Fox J., Shakib F., Martinez-Pomares L. (2011). Recognition of the major cat allergen Fel d 1 through the cysteine-rich domain of the mannose receptor determines its allergenicity. J. Biol. Chem..

[25] Al-Ghouleh A., Johal R., Sharquie I.K., Emara M., Harrington H., Shakib F., Ghaemmaghami A.M. (2012). The glycosylation pattern of common allergens: The recognition and uptake of Der p 1 by epithelial and dendritic cells is carbohydrate dependent. PLoS ONE.

[26] Coughlin S.R. (2000). Thrombin signalling and protease-activated receptors. Nature.

[27] Asokananthan N., Graham P.T., Stewart D.J., Bakker A.J., Eidne K.A., Thompson P.J., Stewart G.A. (2002). House dust mite allergens induce proinflammatory cytokines from respiratory epithelial cells: The cysteine protease allergen, Der p 1, activates protease-activated receptor (PAR)-2 and inactivates PAR-1. J. Immunol..

[28] Sun G., Stacey M.A., Schmidt M., Mori L., Mattoli S. (2001). Interaction of mite allergens Der p3 and Der p9 with protease-activated receptor-2 expressed by lung epithelial cells. J. Immunol..

[29] Tomee J.F., van Weissenbruch R., de Monchy J.G., Kauffman H.F. (1998). Interactions between inhalant allergen extracts and airway epithelial cells: Effect on cytokine production and cell detachment. J. Allergy Clin. Immunol..

[30] King C., Brennan S., Thompson P.J., Stewart G.A. (1998). Dust mite proteolytic allergens induce cytokine release from cultured airway epithelium. J. Immunol..

[31] Kauffman H.F., Tomee J.F., van de Riet M.A., Timmerman A.J., Borger P. (2000). Protease-dependent activation of epithelial cells by fungal allergens leads to morphologic changes and cytokine production. J. Allergy Clin. Immunol..

[32] Yu H.S., Angkasekwinai P., Chang S.H., Chung Y., Dong C. (2010). Protease allergens induce the expression of IL-25 via Erk and p38 MAPK pathway. J. Korean Med. Sci..

[33] Fort M.M., Cheung J., Yen D., Li J., Zurawski S.M., Lo S., Menon S., Clifford T., Hunte B., Lesley R. (2001). IL-25 induces IL-4, IL-5, and IL-13 and Th2-associated pathologies in vivo. Immunity.

[34] Lee J., Ho W.-H., Maruoka M., Corpuz R.T., Baldwin D.T., Foster J.S., Goddard A.D., Yansura D.G., Vandlen R.L., Wood W.I. (2001). IL-17E, a novel proinflammatory ligand for the IL-17 receptor homolog IL-17Rh1. J. Biol. Chem..

[35] Angkasekwinai P., Park H., Wang Y.-H., Wang Y.-H., Chang S.H., Corry D.B., Liu Y.-J., Zhu Z., Dong C. (2007). Interleukin 25 promotes the initiation of proallergic type 2 responses. J. Exp. Med..

[36] Kohanski M.A., Workman A.D., Patel N.N., Hung L.-Y., Shtraks J.P., Chen B., Blasetti M., Doghramji L., Kennedy D.W., Adappa N.D. (2018). Solitary chemosensory cells are a primary epithelial source of IL-25 in patients with chronic rhinosinusitis with nasal polyps. J. Allergy Clin. Immunol..

[37] Von Moltke J., Ji M., Liang H.-E., Locksley R.M. (2016). Tuft-cell-derived IL-25 regulates an intestinal ILC2–epithelial response circuit. Nature.

[38] Corrigan C.J., Wang W., Meng Q., Fang C., Eid G., Caballero M.R., Lv Z., An Y., Wang Y.-H., Liu Y.-J. (2011). Allergen-induced expression of IL-25 and IL-25 receptor in atopic asthmatic airways and late-phase cutaneous responses. J. Allergy Clin. Immunol..

[39] Kang C.-M., Jang A.-S., Ahn M.-H., Shin J.-A., Kim J.-H., Choi Y.-S., Rhim T.-Y., Park C.-S. (2005). Interleukin-25 and interleukin-13 production by alveolar macrophages in response to particles. Am. J. Respir. Cell Mol. Biol..

[40] Wang Y.-H., Angkasekwinai P., Lu N., Voo K.S., Arima K., Hanabuchi S., Hippe A., Corrigan C.J., Dong C., Homey B. (2007). IL-25 augments type 2 immune responses by enhancing the expansion and functions of TSLP-DC–activated Th2 memory cells. J. Exp. Med..

[41] Ikeda K., Nakajima H., Suzuki K., Kagami S.-i., Hirose K., Suto A., Saito Y., Iwamoto I. (2003). Mast cells produce interleukin-25 upon FcεRI-mediated activation. Blood J. Am. Soc. Hematol..

[42] Li Z.-G., Scott M.J., Brzoska T., Sundd P., Li Y.-H., Billiar T.R., Wilson M.A., Wang P., Fan J. (2018). Lung epithelial cell-derived IL-25 negatively regulates LPS-induced exosome release from macrophages. Mil. Med. Res..

[43] Claudio E., Tassi I., Wang H., Tang W., Ha H.L., Siebenlist U. (2015). Cutting Edge: IL-25 Targets Dendritic Cells To Attract IL-9-Producing T Cells in Acute Allergic Lung Inflammation. J. Immunol..

[44] Wang H., Mobini R., Fang Y., Barrenäs F., Zhang H., Xiang Z., Benson M. (2010). Allergen challenge of peripheral blood mononuclear cells from patients with seasonal allergic rhinitis increases IL-17RB, which regulates basophil apoptosis and degranulation. Clin. Exp. Allergy.

[45] Salter B.M., Oliveria J.P., Nusca G., Smith S., Tworek D., Mitchell P., Watson R.M., Sehmi R., Gauvreau G.M. (2016). IL-25 and IL-33 induce Type 2 inflammation in basophils from subjects with allergic asthma. Respir. Res..

[46] Tang W., Smith S.G., Salter B., Oliveria J.P., Mitchell P., Nusca G.M., Howie K., Gauvreau G.M., O’Byrne P.M., Sehmi R. (2016). Allergen-induced increases in interleukin-25 and interleukin-25 receptor expression in mature eosinophils from atopic asthmatics. Int. Arch. Allergy Immunol..

[47] Tang W., Smith S.G., Du W., Gugilla A., Du J., Oliveria J.P., Howie K., Salter B.M., Gauvreau G.M., O’Byrne P.M. (2018). Interleukin-25 and eosinophils progenitor cell mobilization in allergic asthma. Clin. Transl. Allergy.

[48] Wong C.K., Cheung P.F., Ip W.K., Lam C.W. (2005). Interleukin-25–induced chemokines and interleukin-6 release from eosinophils is mediated by p38 mitogen-activated protein kinase, c-Jun N-terminal kinase, and nuclear factor-κB. Am. J. Respir. Cell Mol. Biol..

[49] Cheung P., Wong C., Ip W., Lam C. (2006). IL-25 regulates the expression of adhesion molecules on eosinophils: Mechanism of eosinophilia in allergic inflammation. Allergy.

[50] Furusawa J.-i., Moro K., Motomura Y., Okamoto K., Zhu J., Takayanagi H., Kubo M., Koyasu S. (2013). Critical role of p38 and GATA3 in natural helper cell function. J. Immunol..

[51] Huang Y., Guo L., Qiu J., Chen X., Hu-Li J., Siebenlist U., Williamson P.R., Urban J.F., Paul W.E. (2015). IL-25-responsive, lineage-negative KLRG1 hi cells are multipotential ‘inflammatory’type 2 innate lymphoid cells. Nat. Immunol..

[52] Terashima A., Watarai H., Inoue S., Sekine E., Nakagawa R., Hase K., Iwamura C., Nakajima H., Nakayama T., Taniguchi M. (2008). A novel subset of mouse NKT cells bearing the IL-17 receptor B responds to IL-25 and contributes to airway hyperreactivity. J. Exp. Med..

[53] Flavell R.A., Li B., Dong C., LU H.-T., Yang D.D., Enslen H., Tournier C., Whitmarsh A., Wysk M., Conze D. (1999). Molecular basis of T-cell differentiation. Cold Spring Harb. Symp. Quant. Biol..

[54] Ranger A.M., Hodge M.R., Gravallese E.M., Oukka M., Davidson L., Alt F.W., de la Brousse F.C., Hoey T., Grusby M., Glimcher L.H. (1998). Delayed lymphoid repopulation with defects in IL-4–driven responses produced by inactivation of NF-ATc. Immunity.

[55] Zhu J., Cote-Sierra J., Guo L., Paul W.E. (2003). Stat5 activation plays a critical role in Th2 differentiation. Immunity.

[56] Yao Z., Cui Y., Watford W.T., Bream J.H., Yamaoka K., Hissong B.D., Li D., Durum S.K., Jiang Q., Bhandoola A. (2006). Stat5a/b are essential for normal lymphoid development and differentiation. Proc. Natl. Acad. Sci. USA.

[57] Wu L., Zepp J.A., Qian W., Martin B.N., Ouyang W., Yin W., Bunting K.D., Aronica M., Erzurum S., Li X. (2015). A novel IL-25 signaling pathway through STAT5. J. Immunol..

[58] Angkasekwinai P., Chang S.H., Thapa M., Watarai H., Dong C. (2010). Regulation of IL-9 expression by IL-25 signaling. Nat. Immunol..

[59] Corrigan C.J., Wang W., Meng Q., Fang C., Wu H., Reay V., Lv Z., Fan Y., An Y., Wang Y.-H. (2011). T-helper cell type 2 (Th2) memory T cell-potentiating cytokine IL-25 has the potential to promote angiogenesis in asthma. Proc. Natl. Acad. Sci. USA.

[60] Lajoie-Kadoch S., Joubert P., Létuvé S., Halayko A.J., Martin J.G., Soussi-Gounni A., Hamid Q. (2006). TNF-α and IFN-γ inversely modulate expression of the IL-17E receptor in airway smooth muscle cells. Am. J. Physiol.-Lung Cell. Mol. Physiol..

[61] Létuvé S., Lajoie-Kadoch S., Audusseau S., Rothenberg M.E., Fiset P.-O., Ludwig M.S., Hamid Q. (2006). IL-17E upregulates the expression of proinflammatory cytokines in lung fibroblasts. J. Allergy Clin. Immunol..

[62] Novatchkova M., Leibbrandt A., Werzowa J., Neubüser A., Eisenhaber F. (2003). The STIR-domain superfamily in signal transduction, development and immunity. Trends Biochem. Sci..

[63] Rickel E.A., Siegel L.A., Yoon B.-R.P., Rottman J.B., Kugler D.G., Swart D.A., Anders P.M., Tocker J.E., Comeau M.R., Budelsky A.L. (2008). Identification of functional roles for both IL-17RB and IL-17RA in mediating IL-25-induced activities. J. Immunol..

[64] Xu M., Dong C. (2017). IL-25 in allergic inflammation. Immunol. Rev..

[65] Gaffen S.L. (2009). Structure and signalling in the IL-17 receptor family. Nat. Rev. Immunol..

[66] Iwakura Y., Ishigame H., Saijo S., Nakae S. (2011). Functional specialization of interleukin-17 family members. Immunity.

[67] Chang S.H., Dong C. (2011). Signaling of interleukin-17 family cytokines in immunity and inflammation. Cell. Signal..

[68] Qian Y., Liu C., Hartupee J., Altuntas C.Z., Gulen M.F., Jane-Wit D., Xiao J., Lu Y., Giltiay N., Liu J. (2007). The adaptor Act1 is required for interleukin 17–dependent signaling associated with autoimmune and inflammatory disease. Nat. Immunol..

[69] Claudio E., Sønder S.U., Saret S., Carvalho G., Ramalingam T.R., Wynn T.A., Chariot A., Garcia-Perganeda A., Leonardi A., Paun A. (2009). The adaptor protein CIKS/Act1 is essential for IL-25-mediated allergic airway inflammation. J. Immunol..

[70] Swaidani S., Bulek K., Kang Z., Liu C., Lu Y., Yin W., Aronica M., Li X. (2009). The critical role of epithelial-derived Act1 in IL-17-and IL-25-mediated pulmonary inflammation. J. Immunol..

[71] Bulek K., Liu C., Swaidani S., Wang L., Page R.C., Gulen M.F., Herjan T., Abbadi A., Qian W., Sun D. (2011). The inducible kinase IKKi is required for IL-17-dependent signaling associated with neutrophilia and pulmonary inflammation. Nat. Immunol..

[72] Sun D., Novotny M., Bulek K., Liu C., Li X., Hamilton T. (2011). Treatment with IL-17 prolongs the half-life of chemokine CXCL1 mRNA via the adaptor TRAF5 and the splicing-regulatory factor SF2 (ASF). Nat. Immunol..

[73] Swaidani S., Bulek K., Kang Z., Gulen M.F., Liu C., Yin W., Abbadi A., Aronica M., Li X. (2011). T cell-derived Act1 is necessary for IL-25–mediated Th2 responses and allergic airway inflammation. J. Immunol..

[74] Maezawa Y., Nakajima H., Suzuki K., Tamachi T., Ikeda K., Inoue J.-i., Saito Y., Iwamoto I. (2006). Involvement of TNF receptor-associated factor 6 in IL-25 receptor signaling. J. Immunol..

[75] Swaidani S., Liu C., Zhao J., Bulek K., Li X. (2019). TRAF regulation of IL-17 cytokine signaling. Front. Immunol..

[76] Schwandner R., Yamaguchi K., Cao Z. (2000). Requirement of tumor necrosis factor receptor–associated factor (TRAF) 6 in interleukin 17 signal transduction. J. Exp. Med..

[77] Hartupee J., Liu C., Novotny M., Sun D., Li X., Hamilton T.A. (2009). IL-17 signaling for mRNA stabilization does not require TNF receptor-associated factor 6. J. Immunol..

[78] Zepp J.A., Wu L., Qian W., Ouyang W., Aronica M., Erzurum S., Li X. (2015). TRAF4-SMURF2–Mediated DAZAP2 degradation is critical for IL-25 signaling and allergic airway inflammation. J. Immunol..

[79] Popova A., Kzhyshkowska J., Nurgazieva D., Goerdt S., Gratchev A. (2012). Smurf2 regulates IL17RB by proteasomal degradation of its novel binding partner DAZAP2. Immunobiology.

[80] Monin L., Gaffen S.L. (2018). Interleukin 17 family cytokines: Signaling mechanisms, biological activities, and therapeutic implications. Cold Spring Harb. Perspect. Biol..

[81] Zhu S., Pan W., Shi P., Gao H., Zhao F., Song X., Liu Y., Zhao L., Li X., Shi Y. (2010). Modulation of experimental autoimmune encephalomyelitis through TRAF3-mediated suppression of interleukin 17 receptor signaling. J. Exp. Med..

[82] Wang Y., Zhang Y., Li M.-Q., Fan D.-X., Wang X.-H., Li D.-J., Jin L.-P. (2014). Interleukin-25 induced by human chorionic gonadotropin promotes the proliferation of decidual stromal cells by activation of JNK and AKT signal pathways. Fertil. Steril..

[83] Luo Y., Yang Z., Su L., Shan J., Xu H., Xu Y., Liu L., Zhu W., Chen X., Liu C. (2016). Non-CSCs nourish CSCs through interleukin-17E-mediated activation of NF-κB and JAK/STAT3 signaling in human hepatocellular carcinoma. Cancer Lett..

[84] Saharinen P., Ekman N., Sarvas K., Parker P., Alitalo K., Silvennoinen O. (1997). The Bmx tyrosine kinase induces activation of the Stat signaling pathway, which is specifically inhibited by protein kinase Cδ. Blood J. Am. Soc. Hematol..

[85] Wu H.-H., Hwang-Verslues W.W., Lee W.-H., Huang C.-K., Wei P.-C., Chen C.-L., Shew J.-Y., Lee E.Y.-H., Jeng Y.-M., Tien Y.-W. (2015). Targeting IL-17B–IL-17RB signaling with an anti–IL-17RB antibody blocks pancreatic cancer metastasis by silencing multiple chemokines. J. Exp. Med..

[86] Cheng D., Xue Z., Yi L., Shi H., Zhang K., Huo X., Bonser L.R., Zhao J., Xu Y., Erle D.J. (2014). Epithelial interleukin-25 is a key mediator in Th2-high, corticosteroid-responsive asthma. Am. J. Respir. Crit. Care Med..

[87] Wang W., Li Y., Lv Z., Chen Y., Huang K., Corrigan C.J., Ying S. (2018). Bronchial Allergen Challenge of Patients with Atopic Asthma Triggers an Alarmin (IL-33, TSLP, and IL-25) Response in the Airways Epithelium and Submucosa. J. Immunol..

[88] Tworek D., Smith S.G., Salter B.M., Baatjes A.J., Scime T., Watson R., Obminski C., Gauvreau G.M., O’Byrne P.M. (2016). IL-25 receptor expression on airway dendritic cells after allergen challenge in subjects with asthma. Am. J. Respir. Crit. Care Med..

[89] Han M., Rajput C., Hong J.Y., Lei J., Hinde J.L., Wu Q., Bentley J.K., Hershenson M.B. (2017). The Innate Cytokines IL-25, IL-33, and TSLP Cooperate in the Induction of Type 2 Innate Lymphoid Cell Expansion and Mucous Metaplasia in Rhinovirus-Infected Immature Mice. J. Immunol..

[90] Ballantyne S.J., Barlow J.L., Jolin H.E., Nath P., Williams A.S., Chung K.F., Sturton G., Wong S.H., McKenzie A.N. (2007). Blocking IL-25 prevents airway hyperresponsiveness in allergic asthma. J. Allergy Clin. Immunol..

[91] Busse W.W., Holgate S., Kerwin E., Chon Y., Feng J., Lin J., Lin S.L. (2013). Randomized, double-blind, placebo-controlled study of brodalumab, a human anti-IL-17 receptor monoclonal antibody, in moderate to severe asthma. Am. J. Respir. Crit. Care Med..

[92] Carriere V., Roussel L., Ortega N., Lacorre D.-A., Americh L., Aguilar L., Bouche G., Girard J.-P. (2007). IL-33, the IL-1-like cytokine ligand for ST2 receptor, is a chromatin-associated nuclear factor in vivo. Proc. Natl. Acad. Sci. USA.

[93] Moussion C., Ortega N., Girard J.-P. (2008). The IL-1-like cytokine IL-33 is constitutively expressed in the nucleus of endothelial cells and epithelial cells in vivo: A novel ‘alarmin’?. PLoS ONE.

[94] Meephansan J., Tsuda H., Komine M., Tominaga S.-i., Ohtsuki M. (2012). Regulation of IL-33 expression by IFN-γ and tumor necrosis factor-α in normal human epidermal keratinocytes. J. Investig. Dermatol..

[95] Préfontaine D., Nadigel J., Chouiali F., Audusseau S., Semlali A., Chakir J., Martin J.G., Hamid Q. (2010). Increased IL-33 expression by epithelial cells in bronchial asthma. J. Allergy Clin. Immunol..

[96] Préfontaine D., Lajoie-Kadoch S., Foley S., Audusseau S., Olivenstein R., Halayko A.J., Lemière C., Martin J.G., Hamid Q. (2009). Increased expression of IL-33 in severe asthma: Evidence of expression by airway smooth muscle cells. J. Immunol..

[97] Savinko T., Matikainen S., Saarialho-Kere U., Lehto M., Wang G., Lehtimäki S., Karisola P., Reunala T., Wolff H., Lauerma A. (2012). IL-33 and ST2 in atopic dermatitis: Expression profiles and modulation by triggering factors. J. Investig. Dermatol..

[98] Roussel L., Erard M., Cayrol C., Girard J.P. (2008). Molecular mimicry between IL-33 and KSHV for attachment to chromatin through the H2A–H2B acidic pocket. EMBO Rep..

[99] Drake L.Y., Kita H. (2017). IL-33: Biological properties, functions, and roles in airway disease. Immunol. Rev..

[100] Bessa J., Meyer C.A., de Vera Mudry M.C., Schlicht S., Smith S.H., Iglesias A., Cote-Sierra J. (2014). Altered subcellular localization of IL-33 leads to non-resolving lethal inflammation. J. Autoimmun..

[101] Schmitz J., Owyang A., Oldham E., Song Y., Murphy E., McClanahan T.K., Zurawski G., Moshrefi M., Qin J., Li X. (2005). IL-33, an interleukin-1-like cytokine that signals via the IL-1 receptor-related protein ST2 and induces T helper type 2-associated cytokines. Immunity.

[102] Coyle A.J., Lloyd C., Tian J., Nguyen T., Erikkson C., Wang L., Ottoson P., Persson P., Delaney T., Lehar S. (1999). Crucial role of the interleukin 1 receptor family member T1/ST2 in T helper cell type 2–mediated lung mucosal immune responses. J. Exp. Med..

[103] Löhning M., Stroehmann A., Coyle A.J., Grogan J.L., Lin S., Gutierrez-Ramos J.-C., Levinson D., Radbruch A., Kamradt T. (1998). T1/ST2 is preferentially expressed on murine Th2 cells, independent of interleukin 4, interleukin 5, and interleukin 10, and important for Th2 effector function. Proc. Natl. Acad. Sci. USA.

[104] Tominaga S.-i., Jenkins N.A., Gilbert D.J., Copeland N.G., Tetsuka T. (1991). Molecular cloning of the murine ST2 gene. Characterization and chromosomal mapping. Biochim. Et Biophys. Acta (BBA)-Gene Struct. Expr..

[105] Yanagisawa K., Takagi T., Tsukamoto T., Tetsuka T., Tominaga S.-i. (1993). Presence of a novel primary response gene ST2L, encoding a product highly similar to the interleukin 1 receptor type 1. FEBS Lett..

[106] Hayakawa H., Hayakawa M., Kume A., Tominaga S.-i. (2007). Soluble ST2 blocks interleukin-33 signaling in allergic airway inflammation. J. Biol. Chem..

[107] Hayakawa H., Hayakawa M., Tominaga S.-i. (2016). Soluble ST2 suppresses the effect of interleukin-33 on lung type 2 innate lymphoid cells. Biochem. Biophys. Rep..

[108] Joshi A.D., Oak S.R., Hartigan A.J., Finn W.G., Kunkel S.L., Duffy K.E., Das A., Hogaboam C.M. (2010). Interleukin-33 contributes to both M1 and M2 chemokine marker expression in human macrophages. BMC Immunol..

[109] Kurowska-Stolarska M., Stolarski B., Kewin P., Murphy G., Corrigan C.J., Ying S., Pitman N., Mirchandani A., Rana B., van Rooijen N. (2009). IL-33 amplifies the polarization of alternatively activated macrophages that contribute to airway inflammation. J. Immunol..

[110] Ashlin T.G., Buckley M.L., Salter R.C., Johnson J.L., Kwan A.P., Ramji D.P. (2014). The anti-atherogenic cytokine interleukin-33 inhibits the expression of a disintegrin and metalloproteinase with thrombospondin motifs-1,-4 and-5 in human macrophages: Requirement of extracellular signal-regulated kinase, c-Jun N-terminal kinase and phosphoinositide 3-kinase signaling pathways. Int. J. Biochem. Cell Biol..

[111] Besnard A.G., Togbe D., Guillou N., Erard F., Quesniaux V., Ryffel B. (2011). IL-33-activated dendritic cells are critical for allergic airway inflammation. Eur. J. Immunol..

[112] Ro M., Lee A.J., Kim J.H. (2018). 5-/12-Lipoxygenase-linked cascade contributes to the IL-33-induced synthesis of IL-13 in mast cells, thus promoting asthma development. Allergy.

[113] Pecaric-Petkovic T., Didichenko S.A., Kaempfer S., Spiegl N., Dahinden C.A. (2009). Human basophils and eosinophils are the direct target leukocytes of the novel IL-1 family member IL-33. Blood J. Am. Soc. Hematol..

[114] Liu T., Barrett N.A., Kanaoka Y., Yoshimoto E., Garofalo D., Cirka H., Feng C., Boyce J.A. (2018). Type 2 Cysteinyl Leukotriene Receptors Drive IL-33–Dependent Type 2 Immunopathology and Aspirin Sensitivity. J. Immunol..

[115] Eiwegger T., Akdis C.A. (2011). IL-33 links tissue cells, dendritic cells and Th2 cell development in a mouse model of asthma. Eur. J. Immunol..

[116] Rank M.A., Kobayashi T., Kozaki H., Bartemes K.R., Squillace D.L., Kita H. (2009). IL-33–activated dendritic cells induce an atypical TH2-type response. J. Allergy Clin. Immunol..

[117] Jung M.-Y., Smrž D., Desai A., Bandara G., Ito T., Iwaki S., Kang J.-H., Andrade M.V., Hilderbrand S.C., Brown J.M. (2013). IL-33 induces a hyporesponsive phenotype in human and mouse mast cells. J. Immunol..

[118] Drube S., Kraft F., Dudeck J., Müller A.-L., Weber F., Göpfert C., Meininger I., Beyer M., Irmler I., Häfner N. (2016). MK2/3 are pivotal for IL-33–induced and mast cell–dependent leukocyte recruitment and the resulting skin inflammation. J. Immunol..

[119] Smithgall M.D., Comeau M.R., Park Yoon B.-R., Kaufman D., Armitage R., Smith D.E. (2008). IL-33 amplifies both Th1-and Th2-type responses through its activity on human basophils, allergen-reactive Th2 cells, iNKT and NK cells. Int. Immunol..

[120] Rivellese F., Suurmond J., de Paulis A., Marone G., Huizinga T.W., Toes R.E. (2014). IgE and IL-33− mediated triggering of human basophils inhibits TLR4− induced monocyte activation. Eur. J. Immunol..

[121] Blom L., Poulsen B.C., Jensen B.M., Hansen A., Poulsen L.K. (2011). IL-33 induces IL-9 production in human CD4+ T cells and basophils. PLoS ONE.

[122] Suzukawa M., Iikura M., Koketsu R., Nagase H., Tamura C., Komiya A., Nakae S., Matsushima K., Ohta K., Yamamoto K. (2008). An IL-1 cytokine member, IL-33, induces human basophil activation via its ST2 receptor. J. Immunol..

[123] Bouffi C., Rochman M., Zust C.B., Stucke E.M., Kartashov A., Fulkerson P.C., Barski A., Rothenberg M.E. (2013). IL-33 markedly activates murine eosinophils by an NF-κB–dependent mechanism differentially dependent upon an IL-4–driven autoinflammatory loop. J. Immunol..

[124] Suzukawa M., Koketsu R., Iikura M., Nakae S., Matsumoto K., Nagase H., Saito H., Matsushima K., Ohta K., Yamamoto K. (2008). Interleukin-33 enhances adhesion, CD11b expression and survival in human eosinophils. Lab. Investig..

[125] Cherry W.B., Yoon J., Bartemes K.R., Iijima K., Kita H. (2008). A novel IL-1 family cytokine, IL-33, potently activates human eosinophils. J. Allergy Clin. Immunol..

[126] Halim T.Y., Rana B.M., Walker J.A., Kerscher B., Knolle M.D., Jolin H.E., Serrao E.M., Haim-Vilmovsky L., Teichmann S.A., Rodewald H.-R. (2018). Tissue-restricted adaptive type 2 immunity is orchestrated by expression of the costimulatory molecule OX40L on group 2 innate lymphoid cells. Immunity.

[127] Chen R., Smith S.G., Salter B., El-Gammal A., Oliveria J.P., Obminski C., Watson R., O’Byrne P.M., Gauvreau G.M., Sehmi R. (2017). Allergen-induced increases in sputum levels of group 2 innate lymphoid cells in subjects with asthma. Am. J. Respir. Crit. Care Med..

[128] Yew Liew F., Girard J., Roderick Turnquist H. (2016). Interleukin-33 in health and disease. Nat. Immunol..

[129] Salmond R.J., Mirchandani A.S., Besnard A.-G., Bain C.C., Thomson N.C., Liew F.Y. (2012). IL-33 induces innate lymphoid cell–mediated airway inflammation by activating mammalian target of rapamycin. J. Allergy Clin. Immunol..

[130] Funakoshi-Tago M., Tago K., Sato Y., Tominaga S.-i., Kasahara T. (2011). JAK2 is an important signal transducer in IL-33-induced NF-κB activation. Cell. Signal..

[131] Endo Y., Hirahara K., Iinuma T., Shinoda K., Tumes D.J., Asou H.K., Matsugae N., Obata-Ninomiya K., Yamamoto H., Motohashi S. (2015). The interleukin-33-p38 kinase axis confers memory T helper 2 cell pathogenicity in the airway. Immunity.

[132] Yamamoto T., Endo Y., Onodera A., Hirahara K., Asou H.K., Nakajima T., Kanno T., Ouchi Y., Uematsu S., Nishimasu H. (2018). DUSP10 constrains innate IL-33-mediated cytokine production in ST2 hi memory-type pathogenic Th2 cells. Nat. Commun..

[133] Yagami A., Orihara K., Morita H., Futamura K., Hashimoto N., Matsumoto K., Saito H., Matsuda A. (2010). IL-33 mediates inflammatory responses in human lung tissue cells. J. Immunol..

[134] Lingel A., Weiss T.M., Niebuhr M., Pan B., Appleton B.A., Wiesmann C., Bazan J.F., Fairbrother W.J. (2009). Structure of IL-33 and its interaction with the ST2 and IL-1RAcP receptors—insight into heterotrimeric IL-1 signaling complexes. Structure.

[135] Liu X., Hammel M., He Y., Tainer J.A., Jeng U.-S., Zhang L., Wang S., Wang X. (2013). Structural insights into the interaction of IL-33 with its receptors. Proc. Natl. Acad. Sci. USA.

[136] Mun S.H., Ko N.Y., Kim H.S., Kim J.W., Kim A.-R., Lee S.H., Kim Y.-G., Lee C.K., Lee S.H., Kim B.K. (2010). Interleukin-33 stimulates formation of functional osteoclasts from human CD14+ monocytes. Cell. Mol. Life Sci..

[137] Funakoshi-Tago M., Tago K., Hayakawa M., Tominaga S.-i., Ohshio T., Sonoda Y., Kasahara T. (2008). TRAF6 is a critical signal transducer in IL-33 signaling pathway. Cell. Signal..

[138] Al-Sajee D., Sehmi R., Hawke T.J., El-Gammal A., Howie K.J., Watson R.M., Londei M., Gauvreau G.M., O’Byrne P.M. (2018). Expression of IL-33 and TSLP and Their Receptors in Asthmatic Airways after Inhaled Allergen Challenge. Am. J. Respir. Crit. Care Med..

[139] Cayrol C., Duval A., Schmitt P., Roga S., Camus M., Stella A., Burlet-Schiltz O., Gonzalez-de-Peredo A., Girard J.P. (2018). Environmental allergens induce allergic inflammation through proteolytic maturation of IL-33. Nat. Immunol..

[140] Hesse L., van Ieperen N., Habraken C., Petersen A.H., Korn S., Smilda T., Goedewaagen B., Ruiters M.H., van der Graaf A.C., Nawijn M.C. (2018). Subcutaneous immunotherapy with purified Der p1 and 2 suppresses type 2 immunity in a murine asthma model. Allergy.

[141] Kaur D., Gomez E., Doe C., Berair R., Woodman L., Saunders R., Hollins F., Rose F., Amrani Y., May R. (2015). IL-33 drives airway hyper-responsiveness through IL-13-mediated mast cell: Airway smooth muscle crosstalk. Allergy.

[142] Li Y., Wang W., Lv Z., Li Y., Chen Y., Huang K., Corrigan C.J., Ying S. (2018). Elevated expression of IL-33 and TSLP in the airways of human asthmatics in vivo: A potential biomarker of severe refractory disease. J. Immunol..

[143] Momen T., Ahanchian H., Reisi M., Shamsdin S.A., Shahsanai A., Keivanfar M. (2017). Comparison of interleukin-33 serum levels in asthmatic patients with a control group and relation with the severity of the disease. Int. J. Prev. Med..

[144] Lachowicz-Scroggins M., Woodruff P., Fahy J., Gordon E. (2013). Characterization of IL-33 and St2 expression in human asthma. Am. J. Respir. Crit. Care Med..

[145] Mitchell P.D., Salter B.M., Oliveria J.P., El-Gammal A., Tworek D., Smith S.G., Sehmi R., Gauvreau G.M., O’Byrne P.M. (2018). IL-33 and its receptor ST2 after inhaled allergen challenge in allergic asthmatics. Int. Arch. Allergy Immunol..

[146] Hardman C., Ogg G. (2016). Interleukin-33, friend and foe in type-2 immune responses. Curr. Opin. Immunol..

[147] Wechsler M.E., Ruddy M.K., Pavord I.D., Israel E., Rabe K.F., Ford L.B., Maspero J.F., Abdulai R.M., Hu C.C., Martincova R. (2021). Efficacy and Safety of Itepekimab in Patients with Moderate-to-Severe Asthma. N. Engl. J. Med..

[148] Kelsen S.G., Agache I.O., Soong W., Israel E., Chupp G.L., Cheung D.S., Theess W., Yang X., Staton T.L., Choy D.F. (2021). Astegolimab (anti-ST2) efficacy and safety in adults with severe asthma: A randomized clinical trial. J. Allergy Clin. Immunol..

[149] Dong H., Hu Y., Liu L., Zou M., Huang C., Luo L., Yu C., Wan X., Zhao H., Chen J. (2016). Distinct roles of short and long thymic stromal lymphopoietin isoforms in house dust mite-induced asthmatic airway epithelial barrier disruption. Sci. Rep..

[150] Fornasa G., Tsilingiri K., Caprioli F., Botti F., Mapelli M., Meller S., Kislat A., Homey B., Di Sabatino A., Sonzogni A. (2015). Dichotomy of short and long thymic stromal lymphopoietin isoforms in inflammatory disorders of the bowel and skin. J. Allergy Clin. Immunol..

[151] Harada M., Hirota T., Jodo A.I., Doi S., Kameda M., Fujita K., Miyatake A., Enomoto T., Noguchi E., Yoshihara S. (2009). Functional analysis of the thymic stromal lymphopoietin variants in human bronchial epithelial cells. Am. J. Respir. Cell Mol. Biol..

[152] Poposki J.A., Klingler A.I., Stevens W.W., Peters A.T., Hulse K.E., Grammer L.C., Schleimer R.P., Welch K.C., Smith S.S., Sidle D.M. (2017). Proprotein convertases generate a highly functional heterodimeric form of thymic stromal lymphopoietin in humans. J. Allergy Clin. Immunol..

[153] Allakhverdi Z., Comeau M.R., Jessup H.K., Yoon B.-R.P., Brewer A., Chartier S., Paquette N., Ziegler S.F., Sarfati M., Delespesse G. (2007). Thymic stromal lymphopoietin is released by human epithelial cells in response to microbes, trauma, or inflammation and potently activates mast cells. J. Exp. Med..

[154] Kato A., Favoreto S., Avila P.C., Schleimer R.P. (2007). TLR3-and Th2 cytokine-dependent production of thymic stromal lymphopoietin in human airway epithelial cells. J. Immunol..

[155] Vu A.T., Baba T., Chen X., Le T.A., Kinoshita H., Xie Y., Kamijo S., Hiramatsu K., Ikeda S., Ogawa H. (2010). Staphylococcus aureus membrane and diacylated lipopeptide induce thymic stromal lymphopoietin in keratinocytes through the Toll-like receptor 2–Toll-like receptor 6 pathway. J. Allergy Clin. Immunol..

[156] Soumelis V., Reche P.A., Kanzler H., Yuan W., Edward G., Homey B., Gilliet M., Ho S., Antonenko S., Lauerma A. (2002). Human epithelial cells trigger dendritic cell–mediated allergic inflammation by producing TSLP. Nat. Immunol..

[157] Zhang K., Shan L., Rahman M.S., Unruh H., Halayko A.J., Gounni A.S. (2007). Constitutive and inducible thymic stromal lymphopoietin expression in human airway smooth muscle cells: Role in chronic obstructive pulmonary disease. Am. J. Physiol.-Lung Cell. Mol. Physiol..

[158] Okayama Y., Okumura S., Sagara H., Yuki K., Sasaki T., Watanabe N., Fueki M., Sugiyama K., Takeda K., Fukuda T. (2009). FcϵRI-mediated thymic stromal lymphopoietin production by interleukin-4-primed human mast cells. Eur. Respir. J..

[159] Allakhverdi Z., Comeau M.R., Jessup H.K., Delespesse G. (2009). Thymic stromal lymphopoietin as a mediator of crosstalk between bronchial smooth muscles and mast cells. J. Allergy Clin. Immunol..

[160] Nomura K., Kojima T., Fuchimoto J., Obata K., Keira T., Himi T., Sawada N. (2012). Regulation of interleukin-33 and thymic stromal lymphopoietin in human nasal fibroblasts by proinflammatory cytokines. Laryngoscope.

[161] Kashyap M., Rochman Y., Spolski R., Samsel L., Leonard W.J. (2011). Thymic stromal lymphopoietin is produced by dendritic cells. J. Immunol..

[162] Li Y.J. (2007). Thymic stromal lymphopoietin and OX40 ligand pathway in the initiation of dendritic cell-mediated allergic inflammation. J. Allergy. Clin. Immunol..

[163] Pandey A., Ozaki K., Baumann H., Levin S.D., Puel A., Farr A.G., Ziegler S.F., Leonard W.J., Lodish H.F. (2000). Cloning of a receptor subunit required for signaling by thymic stromal lymphopoietin. Nat. Immunol..

[164] Park L.S., Martin U., Garka K., Gliniak B., Di Santo J.P., Muller W., Largaespada D.A., Copeland N.G., Jenkins N.A., Farr A.G. (2000). Cloning of the murine thymic stromal lymphopoietin (TSLP) receptor: Formation of a functional heteromeric complex requires interleukin 7 receptor. J. Exp. Med..

[165] Hirano R., Hasegawa S., Hashimoto K., Haneda Y., Ohsaki A., Ichiyama T. (2011). Human thymic stromal lymphopoietin enhances expression of CD80 in human CD14+ monocytes/macrophages. Inflamm. Res..

[166] Wang Y.-H., Ito T., Wang Y.-H., Homey B., Watanabe N., Martin R., Barnes C.J., McIntyre B.W., Gilliet M., Kumar R. (2006). Maintenance and polarization of human TH2 central memory T cells by thymic stromal lymphopoietin-activated dendritic cells. Immunity.

[167] Siracusa M.C., Saenz S.A., Hill D.A., Kim B.S., Headley M.B., Doering T.A., Wherry E.J., Jessup H.K., Siegel L.A., Kambayashi T. (2011). TSLP promotes interleukin-3-independent basophil haematopoiesis and type 2 inflammation. Nature.

[168] Reche P.A., Soumelis V., Gorman D.M., Clifford T., Liu M.-r., Travis M., Zurawski S.M., Johnston J., Liu Y.-J., Spits H. (2001). Human thymic stromal lymphopoietin preferentially stimulates myeloid cells. J. Immunol..

[169] Han H., Headley M.B., Xu W., Comeau M.R., Zhou B., Ziegler S.F. (2013). Thymic stromal lymphopoietin amplifies the differentiation of alternatively activated macrophages. J. Immunol..

[170] Froidure A., Shen C., Gras D., Van Snick J., Chanez P., Pilette C. (2014). Myeloid dendritic cells are primed in allergic asthma for thymic stromal lymphopoietin-mediated induction of Th2 and Th9 responses. Allergy.

[171] Watanabe N., Hanabuchi S., Soumelis V., Yuan W., Ho S., de Waal Malefyt R., Liu Y.-J. (2004). Human thymic stromal lymphopoietin promotes dendritic cell–mediated CD4+ T cell homeostatic expansion. Nat. Immunol..

[172] Arima K., Watanabe N., Hanabuchi S., Chang M., Sun S.-C., Liu Y.-J. (2010). Distinct signal codes generate dendritic cell functional plasticity. Sci. Signal..

[173] Ito T., Wang Y.-H., Duramad O., Hori T., Delespesse G.J., Watanabe N., Qin F., Yao Z., Cao W., Liu Y.-J. (2005). TSLP-activated dendritic cells induce an inflammatory T helper type 2 cell response through OX40 ligand. J. Exp. Med..

[174] Mazzucchelli R., Hixon J.A., Spolski R., Chen X., Li W.Q., Hall V.L., Willette-Brown J., Hurwitz A.A., Leonard W.J., Durum S.K. (2008). Development of regulatory T cells requires IL-7Rα stimulation by IL-7 or TSLP. Blood J. Am. Soc. Hematol..

[175] Lee J.Y., Lim Y.M., Park M.J., Min S.Y., Cho M.L., Sung Y.C., Park S.H., Kim H.Y., Cho Y.G. (2008). Murine thymic stromal lymphopoietin promotes the differentiation of regulatory T cells from thymic CD4+ CD8− CD25− naïve cells in a dendritic cell-independent manner. Immunol. Cell Biol..

[176] Li H., Zhao H., Yu J., Su Y., Cao S., An X., Ren X. (2011). Increased prevalence of regulatory T cells in the lung cancer microenvironment: A role of thymic stromal lymphopoietin. Cancer Immunol. Immunother..

[177] Lu N., Wang Y.-H., Wang Y.-H., Arima K., Hanabuchi S., Liu Y.-J. (2009). TSLP and IL-7 use two different mechanisms to regulate human CD4+ T cell homeostasis. J. Exp. Med..

[178] Rochman Y., Kashyap M., Robinson G.W., Sakamoto K., Gomez-Rodriguez J., Wagner K.-U., Leonard W.J. (2010). Thymic stromal lymphopoietin-mediated STAT5 phosphorylation via kinases JAK1 and JAK2 reveals a key difference from IL-7–induced signaling. Proc. Natl. Acad. Sci. USA.

[179] Rochman Y., Dienger-Stambaugh K., Richgels P.K., Lewkowich I.P., Kartashov A.V., Barski A., Khurana Hershey G.K., Leonard W.J., Singh H. (2018). TSLP signaling in CD4+ T cells programs a pathogenic T helper 2 cell state. Sci. Signal..

[180] Bell B.D., Kitajima M., Larson R.P., Stoklasek T.A., Dang K., Sakamoto K., Wagner K.-U., Kaplan D.H., Reizis B., Hennighausen L. (2013). The transcription factor STAT5 is critical in dendritic cells for the development of TH 2 but not TH 1 responses. Nat. Immunol..

[181] Bleck B., Doris B.T., Gordon T., Ahsan M.R., Reibman J. (2010). Diesel exhaust particle-treated human bronchial epithelial cells upregulate Jagged-1 and OX40 ligand in myeloid dendritic cells via thymic stromal lymphopoietin. J. Immunol..

[182] Kamekura R., Kojima T., Takashima A., Koizumi J.-i., Ogasawara N., Go M., Takano K.-i., Murata M., Tanaka S., Ichimiya S. (2010). Thymic stromal lymphopoietin induces tight junction protein claudin-7 via NF-κB in dendritic cells. Histochem. Cell Biol..

[183] Babina M., Wang Z., Franke K., Zuberbier T. (2021). Thymic Stromal Lymphopoietin Promotes MRGPRX2-Triggered Degranulation of Skin Mast Cells in a STAT5-Dependent Manner with Further Support from JNK. Cells.

[184] Zhang Y., Zhou B. (2012). Functions of thymic stromal lymphopoietin in immunity and disease. Immunol. Res..

[185] Wong C.K., Hu S., Cheung P.F., Lam C.W. (2010). Thymic stromal lymphopoietin induces chemotactic and prosurvival effects in eosinophils: Implications in allergic inflammation. Am. J. Respir. Cell Mol. Biol..

[186] Toki S., Goleniewska K., Zhang J., Zhou W., Newcomb D.C., Zhou B., Kita H., Boyd K.L., Peebles R.S. (2020). TSLP and IL-33 reciprocally promote each other’s lung protein expression and ILC2 receptor expression to enhance innate type-2 airway inflammation. Allergy.

[187] Camelo A., Rosignoli G., Ohne Y., Stewart R.A., Overed-Sayer C., Sleeman M.A., May R.D. (2017). IL-33, IL-25, and TSLP induce a distinct phenotypic and activation profile in human type 2 innate lymphoid cells. Blood Adv..

[188] Wu W.H., Park C.O., Oh S.H., Kim H.J., Kwon Y.S., Bae B.G., Noh J.Y., Lee K.H. (2010). Thymic stromal lymphopoietin–activated invariant natural killer T cells trigger an innate allergic immune response in atopic dermatitis. J. Allergy Clin. Immunol..

[189] Hui C.C., Asher I., Heroux D., Allakhverdi Z., Delespesse G., Denburg J.A. (2011). Effects of thymic stromal lymphopoietin on cord blood progenitor cell differentiation and hemopoietic cytokine receptors expression. Allergy Asthma Clin. Immunol..

[190] Allakhverdi Z., Comeau M.R., Smith D.E., Toy D., Endam L.M., Desrosiers M., Liu Y.-J., Howie K.J., Denburg J.A., Gauvreau G.M. (2009). CD34+ hemopoietic progenitor cells are potent effectors of allergic inflammation. J. Allergy Clin. Immunol..

[191] Levin S.D., Koelling R.M., Friend S.L., Isaksen D.E., Ziegler S.F., Perlmutter R.M., Farr A.G. (1999). Thymic stromal lymphopoietin: A cytokine that promotes the development of IgM+ B cells in vitro and signals via a novel mechanism. J. Immunol..

[192] Friend S.L., Hosier S., Nelson A., Foxworthe D., Williams D., Farr A. (1994). A thymic stromal cell line supports in vitro development of surface IgM+ B cells and produces a novel growth factor affecting B and T lineage cells. Exp. Hematol..

[193] Scheeren F.A., van Lent A.U., Nagasawa M., Weijer K., Spits H., Legrand N., Blom B. (2010). Thymic stromal lymphopoietin induces early human B-cell proliferation and differentiation. Eur. J. Immunol..

[194] Omori M., Ziegler S. (2007). Induction of IL-4 expression in CD4+ T cells by thymic stromal lymphopoietin. J. Immunol..

[195] Rochman I., Watanabe N., Arima K., Liu Y.-J., Leonard W.J. (2007). Cutting edge: Direct action of thymic stromal lymphopoietin on activated human CD4+ T cells. J. Immunol..

[196] Akamatsu T., Watanabe N., Kido M., Saga K., Tanaka J., Kuzushima K., Nishio A., Chiba T. (2008). Human TSLP directly enhances expansion of CD8+ T cells. Clin. Exp. Immunol..

[197] Nguyen K.D., Vanichsarn C., Nadeau K.C. (2010). TSLP directly impairs pulmonary Treg function: Association with aberrant tolerogenic immunity in asthmatic airway. Allergy Asthma Clin. Immunol..

[198] Ying S., O’Connor B., Ratoff J., Meng Q., Mallett K., Cousins D., Robinson D., Zhang G., Zhao J., Lee T.H. (2005). Thymic stromal lymphopoietin expression is increased in asthmatic airways and correlates with expression of Th2-attracting chemokines and disease severity. J. Immunol..

[199] Ying S., O’Connor B., Ratoff J., Meng Q., Fang C., Cousins D., Zhang G., Gu S., Gao Z., Shamji B. (2008). Expression and cellular provenance of thymic stromal lymphopoietin and chemokines in patients with severe asthma and chronic obstructive pulmonary disease. J. Immunol..

[200] Shan L., Redhu N.S., Saleh A., Halayko A.J., Chakir J., Gounni A.S. (2010). Thymic stromal lymphopoietin receptor-mediated IL-6 and CC/CXC chemokines expression in human airway smooth muscle cells: Role of MAPKs (ERK1/2, p38, and JNK) and STAT3 pathways. J. Immunol..

[201] Verstraete K., Peelman F., Braun H., Lopez J., Van Rompaey D., Dansercoer A., Vandenberghe I., Pauwels K., Tavernier J., Lambrecht B.N. (2017). Structure and antagonism of the receptor complex mediated by human TSLP in allergy and asthma. Nat. Commun..

[202] Verstraete K., Van Schie L., Vyncke L., Bloch Y., Tavernier J., Pauwels E., Peelman F., Savvides S.N. (2014). Structural basis of the proinflammatory signaling complex mediated by TSLP. Nat. Struct. Mol. Biol..

[203] Isaksen D.E., Baumann H., Trobridge P.A., Farr A.G., Levin S.D., Ziegler S.F. (1999). Requirement for stat5 in thymic stromal lymphopoietin-mediated signal transduction. J. Immunol..

[204] Quentmeier H., Drexler H., Fleckenstein D., Zaborski M., Armstrong A., Sims J., Lyman S. (2001). Cloning of human thymic stromal lymphopoietin (TSLP) and signaling mechanisms leading to proliferation. Leukemia.

[205] Wohlmann A., Sebastian K., Borowski A., Krause S., Friedrich K. (2010). Signal transduction by the atopy-associated human thymic stromal lymphopoietin (TSLP) receptor depends on Janus kinase function. Biol. Chem..

[206] Liu Y.-J. (2006). Thymic stromal lymphopoietin: Master switch for allergic inflammation. J. Exp. Med..

[207] Pattarini L., Trichot C., Bogiatzi S., Grandclaudon M., Meller S., Keuylian Z., Durand M., Volpe E., Madonna S., Cavani A. (2017). TSLP-activated dendritic cells induce human T follicular helper cell differentiation through OX40-ligand. J. Exp. Med..

[208] Salter B.M., Oliveria J.P., Nusca G., Smith S.G., Watson R.M., Comeau M.l., Sehmi R., Gauvreau G.M. (2015). Thymic stromal lymphopoietin activation of basophils in patients with allergic asthma is IL-3 dependent. J. Allergy Clin. Immunol..

[209] Wang Q., Du J., Zhu J., Yang X., Zhou B. (2015). Thymic stromal lymphopoietin signaling in CD4+ T cells is required for TH2 memory. J. Allergy Clin. Immunol..

[210] Cook E.B., Stahl J.L., Schwantes E.A., Fox K.E., Mathur S.K. (2012). IL-3 and TNFα increase Thymic Stromal Lymphopoietin Receptor (TSLPR) expression on eosinophils and enhance TSLP-stimulated degranulation. Clin. Mol. Allergy.

[211] Zhou B., Comeau M.R., De Smedt T., Liggitt H.D., Dahl M.E., Lewis D.B., Gyarmati D., Aye T., Campbell D.J., Ziegler S.F. (2005). Thymic stromal lymphopoietin as a key initiator of allergic airway inflammation in mice. Nat. Immunol..

[212] Kitajima M., Lee H.C., Nakayama T., Ziegler S.F. (2011). TSLP enhances the function of helper type 2 cells. Eur. J. Immunol..

[213] He R., Oyoshi M.K., Garibyan L., Kumar L., Ziegler S.F., Geha R.S. (2008). TSLP acts on infiltrating effector T cells to drive allergic skin inflammation. Proc. Natl. Acad. Sci. USA.

[214] Ebner S., Forstner M., Wang Y.-H., Wolfram D., Liu Y.-J., Romani N. (2007). Thymic stromal lymphopoietin converts human epidermal Langerhans cells into antigen-presenting cells that induce proallergic T cells. J. Allergy Clin. Immunol..

[215] Yao W., Zhang Y., Jabeen R., Nguyen E.T., Wilkes D.S., Tepper R.S., Kaplan M.H., Zhou B. (2013). Interleukin-9 is required for allergic airway inflammation mediated by the cytokine TSLP. Immunity.

[216] Al-Shami A., Spolski R., Kelly J., Keane-Myers A., Leonard W.J. (2005). A role for TSLP in the development of inflammation in an asthma model. J. Exp. Med..

[217] Liu S., Verma M., Michalec L., Liu W., Sripada A., Rollins D., Good J., Ito Y., Chu H., Gorska M.M. (2018). Steroid resistance of airway type 2 innate lymphoid cells from patients with severe asthma: The role of thymic stromal lymphopoietin. J. Allergy Clin. Immunol..

[218] Miyata M., Hatsushika K., Ando T., Shimokawa N., Ohnuma Y., Katoh R., Suto H., Ogawa H., Masuyama K., Nakao A. (2008). Mast cell regulation of epithelial TSLP expression plays an important role in the development of allergic rhinitis. Eur. J. Immunol..

[219] Sokol C.L., Barton G.M., Farr A.G., Medzhitov R. (2008). A mechanism for the initiation of allergen-induced T helper type 2 responses. Nat. Immunol..

[220] Watanabe N., Wang Y.-H., Lee H.K., Ito T., Wang Y.-H., Cao W., Liu Y.-J. (2005). Hassall’s corpuscles instruct dendritic cells to induce CD4+ CD25+ regulatory T cells in human thymus. Nature.

[221] Milford T.A.M., Su R.J., Francis O.L., Baez I., Martinez S.R., Coats J.S., Weldon A.J., Calderon M.N., Nwosu M.C., Botimer A.R. (2016). TSLP or IL-7 provide an IL-7Rα signal that is critical for human B lymphopoiesis. Eur. J. Immunol..

[222] Gauvreau G.M., O’Byrne P.M., Boulet L.P., Wang Y., Cockcroft D., Bigler J., FitzGerald J.M., Boedigheimer M., Davis B.E., Dias C. (2014). Effects of an anti-TSLP antibody on allergen-induced asthmatic responses. N. Engl. J. Med..

[223] Corren J., Parnes J.R., Wang L., Mo M., Roseti S.L., Griffiths J.M., van der Merwe R. (2017). Tezepelumab in Adults with Uncontrolled Asthma. N. Engl. J. Med..

[224] Menzies-Gow A., Corren J., Bourdin A., Chupp G., Israel E., Griffiths J., Hellqvist Å., Bowen K., Kaur P., Almqvist G. (2021). Efficacy and Safety of Tezepelumab in Adults and Adolescents with Severe, Uncontrolled Asthma: Results from the Phase 3 NAVIGATOR Study. J. Allergy Clin. Immunol..

[225] Huang L., Zhang X., Wang M., Chen Z., Yan Y., Gu W., Tan J., Jiang W., Ji W. (2019). Exosomes from thymic stromal lymphopoietin-activated dendritic cells promote Th2 differentiation through the OX40 ligand. Pathobiology.

[226] Gauvreau G.M., Boulet L.P., Cockcroft D.W., FitzGerald J.M., Mayers I., Carlsten C., Laviolette M., Killian K.J., Davis B.E., Larché M. (2014). OX40L blockade and allergen-induced airway responses in subjects with mild asthma. Clin. Exp. Allergy.

[227] Daya M., Rafaels N., Brunetti T.M., Chavan S., Levin A.M., Shetty A., Gignoux C.R., Boorgula M.P., Wojcik G., Campbell M. (2019). Association study in African-admixed populations across the Americas recapitulates asthma risk loci in non-African populations. Nat. Commun..

[228] Shrine N., Portelli M.A., John C., Soler Artigas M., Bennett N., Hall R., Lewis J., Henry A.P., Billington C.K., Ahmad A. (2019). Moderate-to-severe asthma in individuals of European ancestry: A genome-wide association study. Lancet Respir. Med..

[229] Willis-Owen S.A.G., Cookson W.O.C., Moffatt M.F. (2018). The Genetics and Genomics of Asthma. Annu. Rev. Genom. Hum. Genet..

[230] Hoffjan S., Nicolae D., Ober C. (2003). Association studies for asthma and atopic diseases: A comprehensive review of the literature. Respir. Res..

[231] Matloubi M., Ranjbar M., Assarehzadegan M.-A., Fallahpour M., Sadeghi F., Soleyman-Jahi S., Janani L. (2020). The Impact of Interleukin (IL)-33 Gene Polymorphisms and Environmental Factors on Risk of Asthma in the Iranian Population. Lung.

[232] Tse S.M., Krajinovic M., Chauhan B.F., Zemek R., Gravel J., Chalut D., Poonai N., Quach C., Laberge S., Ducharme F.M. (2019). Genetic determinants of acute asthma therapy response in children with moderate-to-severe asthma exacerbations. Pediatric Pulmonol..

[233] Shaban S.A., Brakhas S.A., Ad’hiah A.H. (2021). Interleukin-33 gene variants (rs928413, rs16924159 and rs7037276) and susceptibility to asthma among Iraqi adult patients. Meta Gene.

[234] Ketelaar M.E., Portelli M.A., Dijk F.N., Shrine N., Faiz A., Vermeulen C.J., Xu C.J., Hankinson J., Bhaker S., Henry A.P. (2021). Phenotypic and functional translation of IL33 genetics in asthma. J. Allergy Clin. Immunol..

[235] Ranjbar M., Matloubi M., Assarehzadegan M.A., Fallahpour M., Sadeghi F., Soleyman-Jahi S., Janani L. (2020). Association between Two Single Nucleotide Polymorphisms of Thymic Stromal Lymphopoietin (TSLP) Gene and Asthma in Iranian Population. Iran. J. Allergy Asthma Immunol..

[236] Elmaraghy M.A., Hodieb M.M., Khattab R.A.E.R., Abdelgalel M.N. (2018). Association between TSLP gene polymorphism and bronchial asthma in children in Beni Suef Governorate in Egypt. Comp. Clin. Pathol..

[237] Alenazy M., Vazquez-Tello A., Kenana R., Sharif-Askari F., Alkahtani A., Jamhawi A., Afzal S., Al-Kufiedy R., Temsah M.-H., Al-Masri A. (2020). Three common single-nucleotide variants in the promoter region of thymic stromal lymphopoietin cytokine are not associated with asthma prevalence in a Saudi Arabian population. J. Nat. Sci. Med..

[238] Sun Y., Wei X., Deng J., Zhang J., He Z., Yang M., Liang S., Chen Z., Qin H. (2019). Association of IL1RL1 rs3771180 and TSLP rs1837253 variants with asthma in the Guangxi Zhuang population in China. J. Clin. Lab. Anal..

[239] Afzal S., Ramzan K., Khan I., Ullah A., Tahir A.I., Ramzan S., Ullah S., Jamal A., Absar M., Basit S. (2020). Thymic Stromal Lymphopoietin (TSLP) gene variant rs1837253 is significantly associated with Asthma prevalence in Pakistani Pashtun women. Pak. J. Pharm. Sci..

[240] Cooper R.S., Tayo B., Zhu X. (2008). Genome-wide association studies: Implications for multiethnic samples. Hum. Mol. Genet..

[241] Hunninghake G.M., Soto-Quirós M.E., Avila L., Kim H.P., Lasky-Su J., Rafaels N., Ruczinski I., Beaty T.H., Mathias R.A., Barnes K.C. (2010). TSLP polymorphisms are associated with asthma in a sex-specific fashion. Allergy.

[242] Han Y.-Y., Forno E., Celedón J.C. (2020). Sex Steroid Hormones and Asthma in a Nationwide Study of U.S. Adults. Am. J. Respir. Crit. Care Med..

[243] Okada H., Kuhn C., Feillet H., Bach J.F. (2010). The ‘hygiene hypothesis’ for autoimmune and allergic diseases: An update. Clin. Exp. Immunol..

[244] Lack G. (2001). Pediatric allergic rhinitis and comorbid disorders. J. Allergy Clin. Immunol..

[245] Kouzaki H., Tojima I., Kita H., Shimizu T. (2013). Transcription of interleukin-25 and extracellular release of the protein is regulated by allergen proteases in airway epithelial cells. Am. J. Respir. Cell Mol. Biol..

[246] Mou Z., Xia J., Tan Y., Wang X., Zhang Y., Zhou B., Li H., Han D. (2009). Overexpression of thymic stromal lymphopoietin in allergic rhinitis. Acta Otolaryngol.

[247] Sakashita M., Yoshimoto T., Hirota T., Harada M., Okubo K., Osawa Y., Fujieda S., Nakamura Y., Yasuda K., Nakanishi K. (2008). Association of serum interleukin-33 level and the interleukin-33 genetic variant with Japanese cedar pollinosis. Clin. Exp. Allergy.

[248] Huang R., Mao W., Wang G., Ding J., Sun Y., Gao G., Dong P., Sun Z. (2020). Synergistic relationship between TSLP and IL-33/ST2 signaling pathways in allergic rhinitis and the effects of hypoxia. Int. Forum Allergy Rhinol..

[249] Wen Y., Zeng Q., Luo X., Ma R., Tang Y., Liu W. (2020). Leptin Promoted IL-17 Production from ILC2s in Allergic Rhinitis. Mediat. Inflamm..

[250] Guttman-Yassky E., Waldman A., Ahluwalia J., Ong P.Y., Eichenfield L.F. (2017). Atopic dermatitis: Pathogenesis. Semin. Cutan. Med. Surg..

[251] Bogiatzi S.I., Fernandez I., Bichet J.C., Marloie-Provost M.A., Volpe E., Sastre X., Soumelis V. (2007). Cutting Edge: Proinflammatory and Th2 cytokines synergize to induce thymic stromal lymphopoietin production by human skin keratinocytes. J. Immunol..

[252] Imai Y., Yasuda K., Sakaguchi Y., Haneda T., Mizutani H., Yoshimoto T., Nakanishi K., Yamanishi K. (2013). Skin-specific expression of IL-33 activates group 2 innate lymphoid cells and elicits atopic dermatitis-like inflammation in mice. Proc. Natl. Acad. Sci. USA.

[253] Suto H., Nambu A., Morita H., Yamaguchi S., Numata T., Yoshizaki T., Shimura E., Arae K., Asada Y., Motomura K. (2018). IL-25 enhances T(H)17 cell-mediated contact dermatitis by promoting IL-1β production by dermal dendritic cells. J. Allergy Clin. Immunol..

[254] Griffiths J., Sridhar S., Gavala M., Pham T.-H., Ohne Y., De Los Reyes M., Pilataxi F., Kagiampakis I., Parnes J., Komjathy S. (2020). Atopic Dermatitis Biomarker Analysis Points to Elevation of TSLP and IL-33 Signaling and Suggests a Role for Type 2 Innate Lymphoid Cells. J. Allergy Clin. Immunol..

[255] Basałygo M., Śliwińska J., Żbikowska-Gotz M., Lis K., Socha E., Nowowiejska L., Bartuzi Z., Zegarska B. (2021). Evaluation of the effect of the interleukin-25 serum concentration on the intensity of the symptoms of atopic dermatitis and epidermal barrier. Postepy Derm. Alergol.

[256] Jaworek A.K., Szafraniec K., Zuber Z., Wojas-Pelc A., Jaworek J. (2020). Interleukin 25, thymic stromal lymphopoietin and house dust mites in pathogenesis of atopic dermatitis. J. Physiol Pharm..

[257] Sicherer S.H., Sampson H.A. (2018). Food allergy: A review and update on epidemiology, pathogenesis, diagnosis, prevention, and management. J. Allergy Clin. Immunol..

[258] Anvari S., Miller J., Yeh C.-Y., Davis C.M. (2019). IgE-Mediated Food Allergy. Clin. Rev. Allergy Immunol..

[259] Ukleja-Sokołowska N., Żbikowska-Gotz M., Lis K., Adamczak R., Bartuzi Z. (2021). Assessment of TSLP, IL 25 and IL 33 in patients with shrimp allergy. Allergy Asthma Clin. Immunol..

[260] Chinthrajah S., Cao S., Liu C., Lyu S.-C., Sindher S.B., Long A., Sampath V., Petroni D., Londei M., Nadeau K.C. (2019). Phase 2a randomized, placebo-controlled study of anti–IL-33 in peanut allergy. JCI Insight.

